# Signal Peptide Hydrophobicity Modulates Interaction with the Twin-Arginine Translocase

**DOI:** 10.1128/mBio.00909-17

**Published:** 2017-08-01

**Authors:** Qi Huang, Tracy Palmer

**Affiliations:** Division of Molecular Microbiology, School of Life Sciences, University of Dundee, Dundee, United Kingdom; NICHD

**Keywords:** Sec pathway, Tat pathway, protein secretion, signal peptide, suppressor genetics

## Abstract

The general secretory pathway (Sec) and twin-arginine translocase (Tat) operate in parallel to export proteins across the cytoplasmic membrane of prokaryotes and the thylakoid membrane of plant chloroplasts. Substrates are targeted to their respective machineries by N-terminal signal peptides that share a tripartite organization; however, Tat signal peptides harbor a conserved and almost invariant arginine pair that is critical for efficient targeting to the Tat machinery. Tat signal peptides interact with a membrane-bound receptor complex comprised of TatB and TatC components, with TatC containing the twin-arginine recognition site. Here, we isolated suppressors in the signal peptide of the Tat substrate, SufI, that restored Tat transport in the presence of inactivating substitutions in the TatC twin-arginine binding site. These suppressors increased signal peptide hydrophobicity, and copurification experiments indicated that they restored binding to the variant TatBC complex. The hydrophobic suppressors could also act in *cis* to suppress substitutions at the signal peptide twin-arginine motif that normally prevent targeting to the Tat pathway. Highly hydrophobic variants of the SufI signal peptide containing four leucine substitutions retained the ability to interact with the Tat system. The hydrophobic signal peptides of two Sec substrates, DsbA and OmpA, containing twin lysine residues, were shown to mediate export by the Tat pathway and to copurify with TatBC. These findings indicate that there is unprecedented overlap between Sec and Tat signal peptides and that neither the signal peptide twin-arginine motif nor the TatC twin-arginine recognition site is an essential mechanistic feature for operation of the Tat pathway.

## IMPORTANCE

Protein export is an essential process in all prokaryotes. The Sec and Tat export pathways operate in parallel, with the Sec machinery transporting unstructured precursors and the Tat pathway transporting folded proteins. Proteins are targeted to the Tat pathway by N-terminal signal peptides that contain an almost invariant twin-arginine motif. Here, we make the surprising discovery that the twin arginines are not essential for recognition of substrates by the Tat machinery and that this requirement can be bypassed by increasing the signal peptide hydrophobicity. We further show that signal peptides of bona fide Sec substrates can also mediate transport by the Tat pathway. Our findings suggest that key features of the Tat targeting mechanism have evolved to prevent mistargeting of substrates to the Sec pathway rather than being a critical requirement for function of the Tat pathway.

## INTRODUCTION

The general secretory (Sec) and twin-arginine translocation (Tat) pathways operate in parallel to transport proteins across the cytoplasmic membranes of prokaryotes and the thylakoid membranes of plant chloroplasts. The Sec pathway translocates substrates in an unfolded conformation, whereas the Tat system transports folded proteins. Many Tat substrates contain redox cofactors that are noncovalently associated, and the Tat system is essential for photosynthesis and some modes of respiratory growth (reviewed in reference 1).

**FIG 1:**
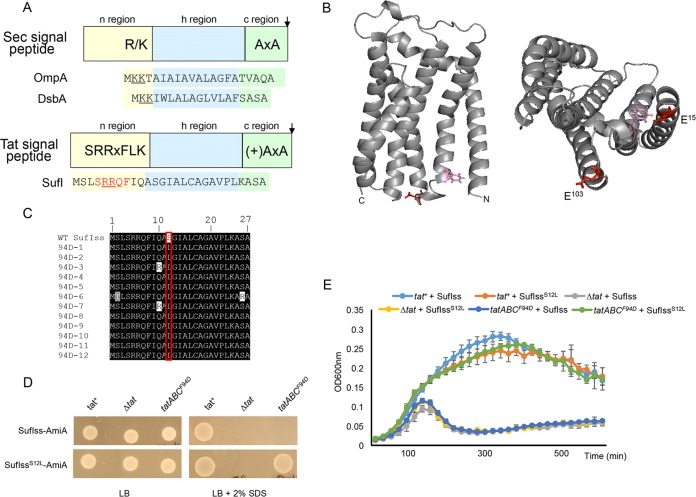
(A) Schematic representation of Sec and Tat signal peptides. The sequences of the OmpA and DsbA Sec-targeting signals and the SufI Tat-targeting signal peptide are shown. Positive charges in the signal peptide n-regions are shown underlined, and the amino acids of the SufI Tat consensus motif are shown in red. (B) Models of *E. coli* TatC. (Left) Side view, with F^94^ and E^103^ residues that are located in the signal peptide binding site given in pink and red, respectively. (Right) View of the cytoplasmic face, with E^15^ additionally shown. (C) Alignment of the amino acid sequence of 12 suppressors in the SufI signal peptide that compensate for the TatC F94D substitution. WT, wild type. (D and E) Cells of strain MC4100 Δ*amiA* Δ*amiC* Δ*tatABC* harboring pTH19kr (empty vector; annotated Δ*tat*) or pTAT101 producing wild-type TatAB along with either wild-type TatC (*tat*^+^) or TatC^F94D^ (*tatABC*^*F94D*^) and a compatible plasmid (either pSUSufIss-mAmiA or pSUSufI^S12L^ss-mAmiA, as indicated) were subcultured at 1:100 into fresh LB medium following overnight growth. (D) Cells were incubated for 3 h at 37°C with shaking. Cells were pelleted and resuspended in sterile phosphate-buffered saline (PBS) supplemented with appropriate antibiotics to an OD_600_ of 0.1, and 8 μl of sample was spotted onto LB agar or LB agar containing 2% SDS. Plates were incubated at 37°C for 16 h. (E) Alternatively, cultures were supplemented with 0.5% SDS (final concentration) and grown at 37°C without shaking. The optical density at 600 nm was monitored every 20 min using a plate reader. Error bars are +standard deviations (*n* = 3 biological replicates).

Targeting of substrates to the Sec and Tat pathways is mediated by the presence of N-terminal signal peptides. Sec and Tat targeting sequences each have a recognizable tripartite structure with a positively charged n-region, a hydrophobic h-region, and a polar c-region that usually contains a cleavage site for leader peptidase (2, 3) (Fig. 1A). One of the primary differences between them is the presence of an almost invariant arginine pair in the n-region of Tat signal peptides. These consecutive arginines are reported to be mechanistically essential for substrate translocation by the Tat pathway, and even conservative alterations to lysine are poorly tolerated (e.g., references 4 and 5). Twin arginines, however, are also compatible with the Sec pathway, and some Sec signal peptides have paired arginines in their n-regions. A second key difference is the relative hydrophobicity of the two types of signal peptide. Tat targeting sequences are notably less hydrophobic than Sec signal peptides, and increasing the hydrophobicity of the TorA signal peptide reroutes a passenger protein from Tat to Sec (6, 7). Finally, one or more positive charges are frequently found in the c-region of Tat signals that are not mechanistically required for Tat transport but serve to block interaction of the signal peptide with the Sec pathway (6–9). Nonetheless, despite these differences, over half of the *Escherichia coli* Tat signal peptides that were tested showed some level of engagement with the Sec pathway when fused to a Sec-compatible reporter protein (10).

Tat signal peptides interact with the membrane-bound Tat receptor complex. In *E. coli*, the receptor contains TatA, TatB, and TatC proteins, most likely in a 1:1:1 stoichiometry (11–13). The receptor is multivalent (14–16) and contains multiple copies of the TatABC heterotrimer (e.g., references 17 and 18). The primary recognition site for the Tat signal peptide is TatC (e.g., references 19 to 23), with two conserved glutamates on the cytoplasmic face of TatC forming a patch that interacts with the signal peptide twin arginines (24) (Fig. 1B). The signal peptide can also transition to a deep binding mode where it is inserted into the receptor complex, forming cross-links to the transmembrane helix (TM) of TatB and TM5 of TatC (17, 19, 25, 26). Signal peptide insertion into the receptor drives structural reorganization of the complex (13, 18, 26, 27) and the recruitment of further TatA molecules (19, 28–30). The assembled TatA oligomer mediates the transport of folded substrates across the membrane in an unknown manner, potentially by forming a translocation channel or by facilitating a localized weakening and transient disruption of the bilayer (31–33).

A recent study isolated genetic suppressors that restored transport activity to a Tat system that harbored an inactivating substitution in the TatC signal peptide binding site (27). These suppressing substitutions, located primarily in the TM of TatB, could also separately restore Tat transport to a substrate with a defective Tat signal peptide. Biochemical analysis revealed that these substitutions did not act to restore detectable signal peptide binding to the receptor complex, but instead, at least some of them induced conformational changes that apparently mimicked the substrate-activated state (27). In this work, we have taken a complementary approach by searching for signal peptide suppressors able to restore Tat transport when the TatC signal peptide binding site was inactivated. We show that two separate inactive TatC variants, F94D and E103K, can be suppressed by single substitutions that increase the hydrophobicity of a Tat signal peptide. Remarkably, the same hydrophobic substitutions can suppress in *cis* by restoring Tat transport to a twin-arginine-mutated signal peptide. Our results show that neither the twin-arginine motif nor its cognate recognition site on TatC is required for Tat transport activity. We further show that hydrophobic Sec signal peptides containing paired lysines can also mediate export by the Tat pathway, pointing to an unexpected degree of overlap between Sec and Tat targeting requirements.

## RESULTS

### Isolation of suppressors of the inactivating TatC F94D substitution.

A series of cross-linking studies, along with direct binding assays using purified TatC variants, have identified that the cytoplasmic N-terminal region and the cytoplasmic loop between TM2 and TM3 form a binding site for the twin-arginine motif of Tat signal peptides (23, 24, 34, 35). Amino acid substitutions in the TM2-TM3 loop in particular are associated with loss of Tat activity, and residues F94 and E103 are almost completely invariant among TatC sequences from all three domains of life (34, 36, 37). Along with E15, E103 has been implicated in coordinating the positively charged twin arginines of the signal peptide (24, 38) (Fig. 1B).

The twin arginines are part of a larger consensus motif, S-R-R-x-F-L-K (Fig. 1A), where the other amino acids are semiconserved (2). The consensus phenylalanine is frequently present, particularly in bacterial Tat signal peptides and, for example, is found in approximately two-thirds of *E. coli* Tat targeting sequences (39). It has been proposed through modeling studies that if the signal peptide n-region is in an extended conformation, TatC^F94^ may stack against this consensus F residue (40). We initially sought to test this hypothesis genetically.

It has been shown previously that a TatC^F94D^ substitution inactivates Tat transport and that strains harboring this substitution are unable to grow on media containing the detergent SDS (27) (Fig. 1D). This phenotype arises due to an inability to export two Tat substrates, AmiA and AmiC, which remodel the cell wall during growth (41, 42). We used a fusion protein whereby the signal peptide of SufI, which has the consensus F residue (Fig. 1A), was fused to the mature region of AmiA (27) and constructed a random library of codon substitutions at F8. We then screened this library against a strain lacking native *amiA*/*amiC* and harboring TatC^F94D^, plating onto LB medium containing 2% SDS to select for suppressors of this inactivating substitution. However, after screening more than 1,000 clones we failed to isolate any suppressors of TatC^F94D^ from this library.

We therefore addressed whether it was possible to isolate substitutions elsewhere in the SufI signal peptide that would suppress TatC^F94D^. To this end, we constructed a random library of mutations throughout the SufI signal peptide coding region of the SufIss-AmiA fusion that had some 13,000 members and an error rate of approximately 2%. After screening more than 20,000 individual transformants for the ability to grow in the presence of 2% SDS, we isolated 12 suppressors that supported growth on the detergent. Sequence analysis indicated that each of the suppressors shared a common alteration of serine at position 12 of the signal peptide to leucine (Fig. 1C), and indeed, this single S12L substitution was sufficient to support growth of a strain producing TatC^F94D^ on LB agar containing SDS (Fig. 1D). Since the phenotypic growth test is largely qualitative, we also undertook a more quantitative assessment of growth of the strain coproducing TatC^F94D^ and SufI^S12L^ss-AmiA by measuring growth curves in the presence of SDS. Figure 1E shows that the strain producing TatC^F94D^ and SufI^S12L^ss-AmiA grew identically to the same strain producing wild-type TatC and SufI^S12L^ss-AmiA.

### The SufI S12L substitution restores transport activity to a different substitution in the TatC signal peptide binding site.

**FIG 2:**
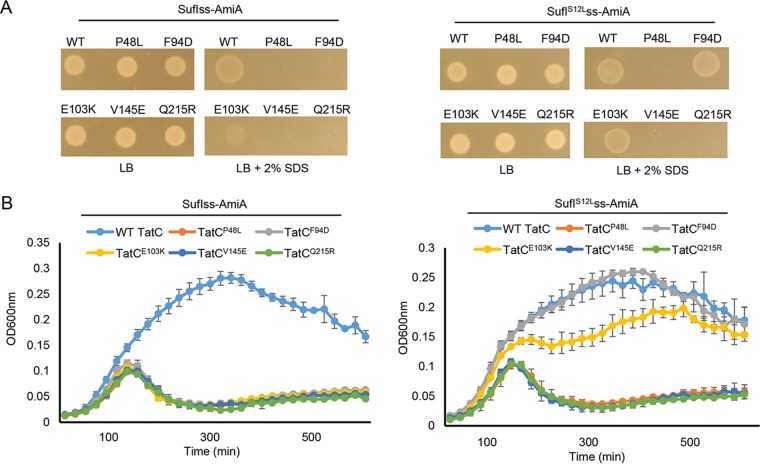
The SufI^S12L^ substitution can restore Tat transport to TatC^E103K^. Overnight cultures of strain MC4100 Δ*amiA* Δ*amiC* Δ*tatABC* harboring either pSUSufIss-mAmiA or pSUSufI^S12L^ss-mAmiA alongside plasmid pTAT101 producing wild-type (WT) TatAB along with the indicated substitution of TatC, as indicated, were subcultured at a 1:100 dilution. (A) Cultures were grown for a further 3 h at 37°C, pelleted, and resuspended to an OD_600_ of 0.1, and 8 μl of sample was spotted onto LB agar or LB agar containing 2% SDS. Plates were incubated at 37°C for 16 h. (B) Alternatively, cultures were supplemented with 0.5% SDS (final concentration) and grown at 37°C without shaking. The optical density at 600 nm was monitored every 20 min using a plate reader. Error bars are +standard deviations (*n* = 3 biological replicates).

To determine whether the suppressor activity of the signal peptide S12L substitution was specific for TatC^F94D^, we tested whether this substitution could restore Tat transport to other TatC-inactivating substitutions, including P48L, V145E, and Q215R, located in consecutive periplasmic loops, or E103K, located in the signal peptide binding site (37). Figure 2 shows that the inactivating TatC^E103K^ substitution could also be suppressed by the SufI^S12L^ variant, but transport activity was not restored to any of the substitutions in the periplasmic loops. We conclude that the S12L substitution specifically restores Tat transport to substitutions in the TatC signal peptide binding site.

### The S12L substitution can restore transport activity to signal peptides that contain inactivating twin-arginine substitutions.

**FIG 3:**
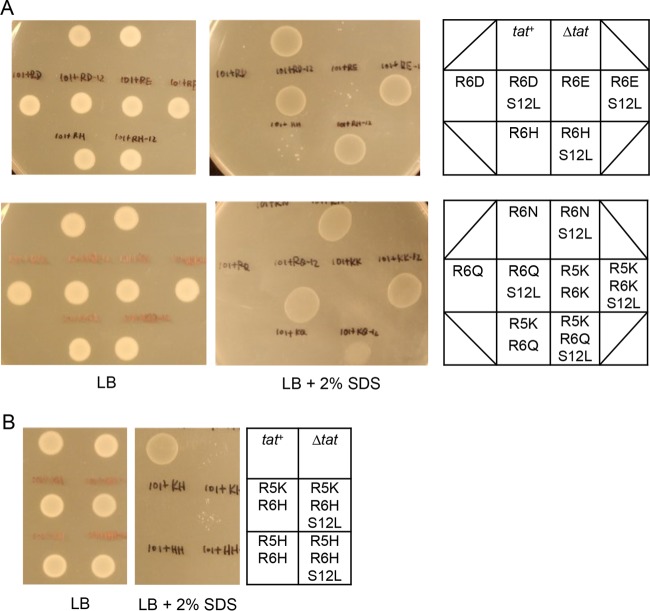
The SufI^S12L^ substitution can act in *cis* to suppress inactivating substitutions in the SufI signal peptide twin-arginine motif. Overnight cultures of strain MC4100 Δ*amiA* Δ*amiC* Δ*tatABC* harboring either pTH19kr alongside pSUSufIss-mAmiA (Δ*tat*) or pTAT101 (producing wild-type TatABC) alongside either unsubstituted pSUSufIss-mAmiA (*tat*^+^) or pSUSufIss-mAmiA encoding the (A) R6D, R6D-S12L, R6E, R6E-S12L, R6H, R6H-S12L, R6N, R6N-S12L, R6Q, R6Q-S12L, R5KR6K, R5KR6K-S12L, R5KR6Q, and R5KR6Q-S12L, or the (B) R5KR6H, R5KR6H-S12L, R5HR6H, and R5HR6H-S12L substitutions in the SufI signal peptide were subcultured at a 1:100 dilution, grown for a further 3 h at 37°C, pelleted, and resuspended to an OD_600_ of 0.1, and 8 μl of sample was spotted onto LB agar or LB agar containing 2% SDS. Plates were incubated at 37°C for 16 h.

Since the signal peptide S12L substitution can act in *trans* to suppress inactivating substitutions in the TatC signal peptide binding site, we next asked whether it could act in *cis* to rescue inactivating substitutions at the twin-arginine motif. Previously, it has been shown that substitutions for one or both consensus arginines of the SufI signal peptide are poorly tolerated (4), and indeed, single mutations of R6 to D, E, H, N, or Q or of R5R6 to KK, KH, KQ, or HH in the SufIss-AmiA fusion are sufficient to prevent growth of cells in the presence of SDS (27) (Fig. 3; also see Fig. S1 in the supplemental material). Interestingly, however, introduction of the S12L substitution alongside R6D, R6E, R6H, R6N, R6Q, or R5K/R6K restored strong growth of cells producing these fusion proteins in the presence of SDS (Fig. 3A and S1B to G). The S12L substitution could also partially compensate for the R5K,R6Q substitution (Fig. 3A and S1H) but could not rescue transport activity of the R5K,R6H or R5H,R6H variants (Fig. 3B and S1I and J). For each of these variant signal peptides, we confirmed that the transport of the AmiA substrate that we observed was dependent on the Tat pathway, since growth on SDS was not observed when the Tat system was absent (Fig. S2). We conclude that the SufI^S12L^ signal peptide substitution can at least partially compensate for substitutions at the twin-arginine motif.

10.1128/mBio.00909-17.1 FIG S1  The SufIS12L substitution can act in *cis* to suppress inactivating substitutions in the SufI signal peptide twin-arginine motif. Overnight cultures of strain MC4100 Δ*amiA* Δ*amiC* Δ*tatABC* harboring pTH19kr alongside pSUSufIss-mAmiA (Δ*tat*) or pTAT101 (producing wild-type TatABC) alongside unsubstituted pSUSufIss-mAmiA (*tat*^+^) (A) or pTAT101 (producing wild-type TatABC) alongside pSUSufIss-mAmiA encoding the indicated substitutions in the SufI signal peptide (B to J) were subcultured at a 1:100 dilution into LB supplemented with 0.5% SDS (final concentration) and grown at 37°C without shaking. The optical density at 600 nm was monitored every 20 min using a plate reader. Error bars are +SD (*n* = 3 biological replicates). Download FIG S1, PDF file, 0.1 MB. Copyright © 2017 Huang and Palmer. 2017 Huang and Palmer This content is distributed under the terms of the Creative Commons Attribution 4.0 International license.

10.1128/mBio.00909-17.2 FIG S2  The twin-arginine substituted SufI^S12L^ signal peptide variants mediate strict Tat-dependent transport of AmiA. (A and B) Overnight cultures of the *tat*^−^ strain MC4100 Δ*amiA* Δ*amiC* Δ*tatABC* harboring either pTAT101 (producing wild-type TatABC) alongside unsubstituted pSUSufIss-mAmiA (*tat*^+^) or pTH19kr alongside either unsubstituted pSUSufIss-mAmiA (Δ*tat*) or pSUSufIss-mAmiA encoding the indicated substitutions in the SufI signal peptide as indicated were subcultured at 1:100 dilution. (A) Cultures were grown for a further 3 h at 37°C, pelleted, and resuspended to an OD_600_ of 0.1, and 8 μl of sample was spotted onto LB agar or LB agar containing 2% SDS. Plates were incubated at 37°C for 16 h. (B) Alternatively, cultures were supplemented with 0.5% SDS (final concentration) and grown at 37°C without shaking. The optical density at 600 nm was monitored every 20 min using a plate reader. Error bars are +SD (*n* = 3 biological replicates). Download FIG S2, PDF file, 0.1 MB. Copyright © 2017 Huang and Palmer. 2017 Huang and Palmer This content is distributed under the terms of the Creative Commons Attribution 4.0 International license.

### Single hydrophobic substitutions along the length of the SufI signal peptide h-region can also suppress inactivating TatC substitutions in the signal peptide binding site.

**FIG 4:**
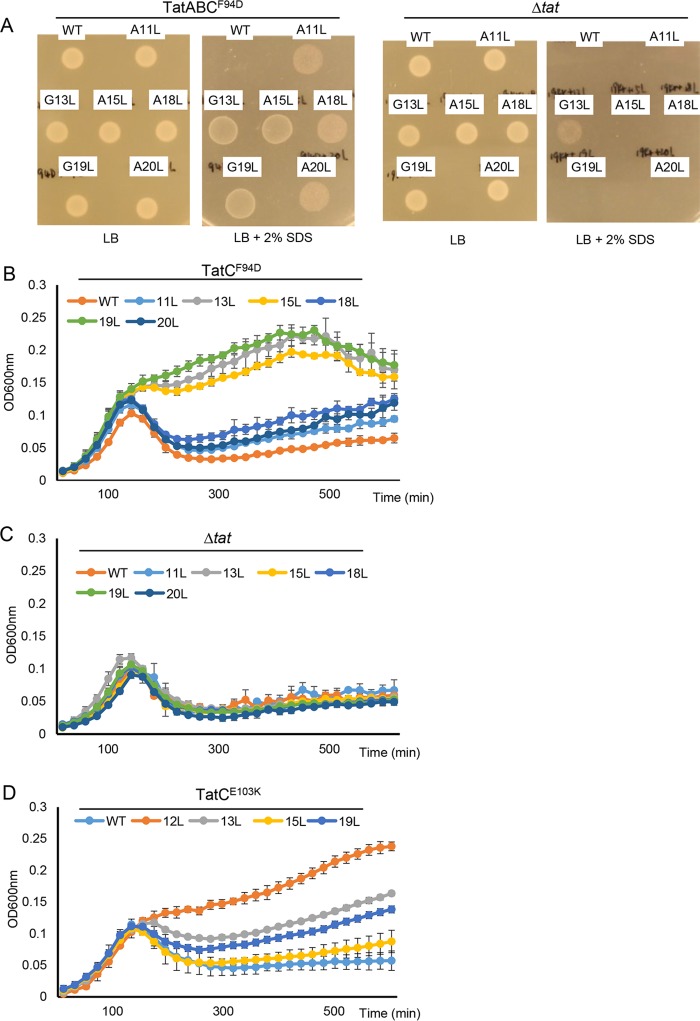
Single leucine substitutions throughout the SufI signal peptide h-region can suppress the TatC F94D and E103K substitutions. (A to C) Overnight cultures of strain MC4100 Δ*amiA* Δ*amiC* Δ*tatABC* harboring either pTH19kr (Δ*tat*) or pTAT101 producing TatABC^F94D^ along with pSUSufIss-mAmiA producing the indicated substitution in the SufI signal peptide were subcultured at a 1:100 dilution. (A) Cultures were grown for a further 3 h at 37°C, pelleted, and resuspended to an OD_600_ of 0.1, and 8 μl of sample was spotted onto LB agar or LB agar containing 2% SDS. Plates were incubated at 37°C for 16 h. (B and C) Alternatively, cultures were supplemented with 0.5% SDS (final concentration) and grown at 37°C without shaking. (D) MC4100 Δ*amiA* Δ*amiC* Δ*tatABC* harboring pTAT101 producing TatABC^E103K^ along with pSUSufIss-mAmiA producing the indicated substitution in the SufI signal peptide was subcultured into LB containing 0.5% SDS and grown at 37°C without shaking. For all growth curves, the optical density at 600 nm was monitored every 20 min using a plate reader. Error bars are +standard deviations (*n* = 3 biological replicates). WT, wild type.

The h-regions of Tat signal peptides are less hydrophobic than Sec signal sequences, containing significantly more glycine and fewer leucine residues (6). The S12L substitution replaces a polar residue near the start of the SufI signal peptide h-region with a highly hydrophobic amino acid, markedly increasing its hydrophobicity score (Table 1). To test whether single hydrophobic substitutions elsewhere in the SufI signal peptide h-region could also suppress Tat transport defects, we increased hydrophobicity of the h-region by constructing individual A11L, G13L, A15L, A18L, G19L, and A20L variants. Figure 4A shows that when each of these individual substitutions was introduced into the SufIss-AmiA construct and produced in a strain harboring *tatC*^*F94D*^, growth on SDS was restored. We assume that we did not obtain any of these substitutions in our original screen because, unlike the S12L substitution, which needed only one nucleotide change, these other substitutions require at least two nucleotide changes.

**TABLE 1:** Relative hydrophobicities of signal peptide variants used in this work^a^

Signal peptide	Sequence	Hydrophobicity
WT SufI	MSLSRRQFIQASGIALCAGAVPLKASA	1.36
SufI 11L	MSLSRRQFIQLSGIALCAGAVPLKASA	1.48
SufI 12L	MSLSRRQFIQALGIALCAGAVPLKASA	1.63
SufI 13L	MSLSRRQFIQASLIALCAGAVPLKASA	1.61
SufI 15L	MSLSRRQFIQASGILLCAGAVPLKASA	1.48
SufI 18L	MSLSRRQFIQASGIALCLGAVPLKASA	1.48
SufI 19L	MSLSRRQFIQASGIALCALAVPLKASA	1.61
SufI 20L	MSLSRRQFIQASGIALCAGLVPLKASA	1.48
SufI 12L,13L	MSLSRRQFIQALLIALCAGAVPLKASA	1.88
SufI 12L,13L,14L,15L	MSLSRRQFIQALLLLLCAGAVPLKASA	1.96
SufI 17L,18L,19L,20L	MSLSRRQFIQASGIALLLLLVPLKASA	1.92
WT OmpA	MKKTAIAIAVALAGFATVAQA	2.31
OmpA i18K	MKKTAIAIAVALAGFAT**K**VAQA	2.31
WT DsbA	MKKIWLALAGLVLAFSASA	2.49
DsbA i16K	MKKIWLALAGLVLAF**K**SASA	2.55

a In each case, the h-region sequence used to calculate the score is shown underlined. For SufI, the signal peptide h-region for each variant was defined using TATFIND 1.4 (62, 63) (http://signalfind.org/tatfind.html). For OmpA and DsbA, the h-region for each variant was defined using Phobius (64) (http://phobius.sbc.su.se/). Hydrophobicity was scored using the grand average of hydropathy (GRAVY) value at http://www.gravy-calculator.de/. WT, wild type. Bold letter K in sequences indicates inserted lysine residue.

When we examined growth on SDS semiquantitatively by monitoring growth curves (Fig. 4B), it could be seen that the G13L, A15L, and G19L substitutions suppressed the *tatC*^*F94D*^ allele more strongly than A11L, A18L, or A20L. Table 1 shows the hydrophobicity scores for the h-regions of these SufI signal peptide variants. It can be seen that the substitutions that give the biggest increase in hydrophobicity result in the strongest level of suppression. It should be noted that the SufIss^G13L^ substitution appeared to result in a very low level of the fusion protein being routed to the Sec pathway, as weak growth could be detected in a strain lacking the Tat pathway (Fig. 4A and C). None of the other substitutions, however, led to any detectable transport by Sec.

We next tested whether these further hydrophobic substitutions could also suppress a second signal peptide binding site substitution, TatC^E103K^. It can be seen (Fig. 4D and S3) that these variants could also compensate for loss of Tat activity resulting from this substitution. They could not, however, compensate for any of the TatC P48L, V145E, and Q215R mutations (not shown). As seen for the suppression of TatC^F94D^, the substitutions giving the biggest increase in hydrophobicity (S12L, G13L, and G19L) restored the highest level of Tat activity in the presence of TatC^E103K^ (Fig. 4D). Finally, we also tested whether the strongest suppressors of TatC^E103K^ could also rescue transport in *cis* when introduced into the twin-lysine variant of the SufI signal peptide. Figure S4 indicates that, similarly to the S12L substitution, introduction of any of the G13L, A15L, and G19L substitutions into KK-SufIss-AmiA restored strong growth of cells producing these fusion proteins in the presence of SDS. We conclude that increasing h-region hydrophobicity can suppress transport defects associated with either the signal peptide twin-arginine motif or the signal peptide binding site.

10.1128/mBio.00909-17.3 FIG S3  Hydrophobic substitutions in the SufI signal peptide can rescue the transport defect of TatC^E103K^. Overnight cultures of strain MC4100 Δ*amiA* Δ*amiC* Δ*tatABC* harboring pTAT101 producing TatABC^E103K^ along with pSUSufIss-mAmiA producing the indicated substitution in the SufI signal peptide were subcultured at a 1:100 dilution, grown for a further 3 h at 37°C, pelleted, and resuspended to an OD_600_ of 0.1, and 8 μl of sample was spotted onto LB agar or LB agar containing 2% SDS. Plates were incubated at 37°C for 16 h. Download FIG S3, PDF file, 0.04 MB. Copyright © 2017 Huang and Palmer. 2017 Huang and Palmer This content is distributed under the terms of the Creative Commons Attribution 4.0 International license.

10.1128/mBio.00909-17.4 FIG S4  Hydrophobic substitutions in the SufI signal peptide can act in *cis* to suppress the signal peptide R5K,R6K substitution. Overnight cultures of MC4100 Δ*amiA* Δ*amiC* Δ*tatABC* harboring pTAT101 (producing wild-type TatABC) alongside either unsubstituted pSUSufIss-mAmiA (WT) or pSUSufIss-mAmiA encoding the indicated substitutions in the SufI signal peptide were subcultured at a 1:100 dilution in the presence of 0.5% SDS (final concentration) and grown at 37°C without shaking. The optical density at 600 nm was monitored every 20 min using a plate reader. Download FIG S4, PDF file, 0.1 MB. Copyright © 2017 Huang and Palmer. 2017 Huang and Palmer This content is distributed under the terms of the Creative Commons Attribution 4.0 International license.

### The h-region suppressors support transport of full-length SufI.

**FIG 5:**
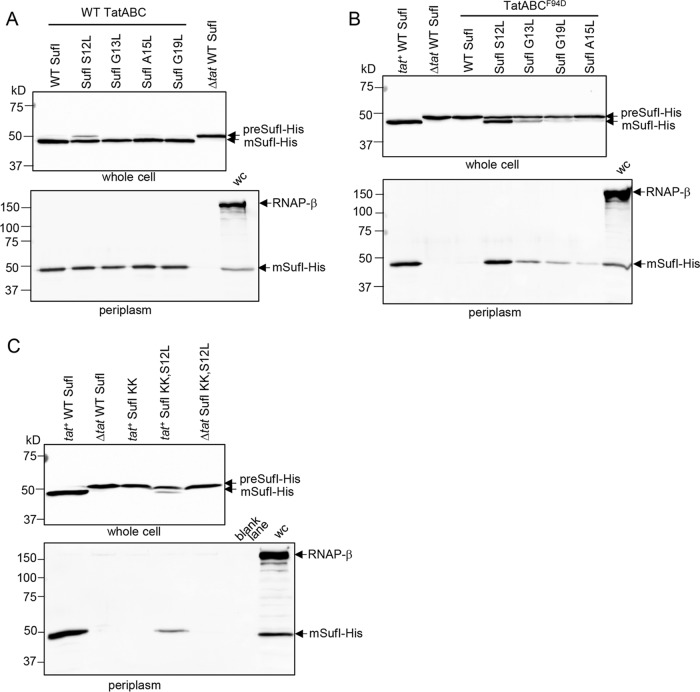
Analysis of SufI export mediated by signal peptide leucine substitutions in the TatC^F94D^ background or when combined with a signal peptide twin-lysine substitution. (A and B) *E. coli* strain DADE coproducing His-tagged but otherwise native SufI or SufI with the indicated single leucine substitutions in the signal peptide (from a pQE80 plasmid) alongside either wild-type (WT) TatABC (A) or wild-type TatAB and TatC^F94D^ (from pTAT101) (B). Strain DADE coproducing His-tagged but otherwise native SufI alongside an empty vector was used as a negative control (lanes annotated “Δ*tat* WT SufI”). (C) Strain DADE producing His-tagged SufI harboring an R5K,R6K double substitution (SufI KK) and with an additional S12L substitution where indicated, alongside either empty vector pTH19kr (Δ*tat*) or pTAT101 encoding wild-type TatABC (*tat*^+^). In each case, strains were grown to mid-log phase and fractionated into whole cell (upper panels) and periplasm (lower panels) and then analyzed by Western blotting with anti-6×His tag or anti-RNA polymerase β-subunit antibodies (cytoplasmic control protein). wc, whole cell. Equivalent volumes of sample were loaded for each of the whole-cell samples and for each of the periplasmic samples.

To assess the level of Tat transport mediated by these hydrophobic variants of the SufI signal sequence, we introduced the S12L, G13L, A15L, and G19L substitutions individually into a construct encoding C-terminally His-tagged but otherwise wild-type SufI. We initially expressed these in a strain producing native TatABC and fractionated cells to obtain the periplasm. Figure 5A shows that each of these single hydrophobic variants of the SufI signal peptide supported strong export of SufI-His. Transport of these SufI-His variants was dependent on the Tat pathway since, with the exception of SufI G13L, where a very faint trace of SufI was seen in the periplasm, no periplasmic SufI-His could be detected when the Tat pathway was absent (Fig. S5A).

10.1128/mBio.00909-17.5 FIG S5  Analysis of SufI export mediated by signal peptide leucine substitutions in the TatC^E103K^ background and in a *tat* deletion strain. *E. coli* strain DADE coproducing His-tagged but otherwise native SufI or SufI with the indicated single leucine substitutions in the signal peptide (from a pQE80 plasmid) alongside either an empty plasmid vector (Δ*tat*) (A) or wild-type TatAB and TatC^E103K^ (from pTAT101) (B) was grown to mid-log phase, fractionated into whole-cell (upper panels) and periplasm (lower panels) samples, and then analyzed by Western blotting with anti-6×His tag or anti-RNA polymerase β-subunit antibodies (cytoplasmic control protein). Strain DADE coproducing His-tagged but otherwise native SufI alongside either wild-type TatABC (*tat*^+^ WT SufI) or an empty vector (Δ*tat* WT SufI) was used as a positive or negative control, respectively. wc, whole cell. Equivalent volumes of sample were loaded for each of the whole-cell samples and for each of the periplasmic samples. Download FIG S5, PDF file, 0.1 MB. Copyright © 2017 Huang and Palmer. 2017 Huang and Palmer This content is distributed under the terms of the Creative Commons Attribution 4.0 International license.

Next, we assessed the degree of transport mediated by these variant signal peptides in cells producing TatABC^F94D^. It can be seen (Fig. 5B) that although wild-type SufI-His was not exported in the presence of TatC^F94D^, transport was detected when any of the single hydrophobic substitutions was present in the signal peptide. The S12L substitution in particular could strongly suppress TatC^F94D^, with high levels of SufI-His detected in the periplasm when the signal peptide harbored this mutation. These same signal peptide substitutions could also restore good transport of SufI-His in the presence of the inactivating TatC^E103K^ substitution (Fig. S5B).

Since the hydrophobic substitutions can act in *cis* to restore transport activity to a twin-lysine variant of the SufI signal peptide twin-arginine motif, we assessed the export of the KK variant of SufI-His harboring the S12L substitution. Figure 5C shows that there was clear Tat transport activity conferred on the twin-lysine signal peptide variant by the presence of the S12L suppressor. Taken together, the results presented so far indicate that the signal peptide consecutive arginines and the TatC twin-arginine recognition site are not essential mechanistic features for operation of the Tat pathway, and substitutions in either of these can be at least partially compensated for by an increase in signal peptide hydrophobicity.

### The h-region suppressors restore signal peptide binding to TatBC.

**FIG 6:**
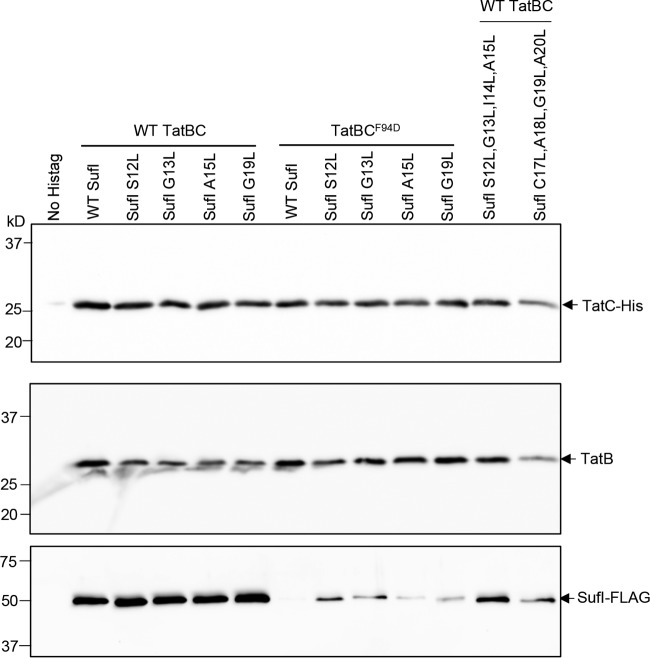
Hydrophobic variants of the SufI signal peptide mediate binding to TatBC and TatBC^F94D^. Cells of strain DADE-P coproducing C-terminally FLAG-tagged SufI with its native signal peptide (WT SufI) or harboring the indicated leucine substitutions, alongside TatB and C-terminally His-tagged TatC or TatC^F94D^, were lysed and incubated with digitonin, and His-tagged TatC was isolated with Ni-charged beads. Following elution of bound TatC-His, equivalent volumes of the eluate from each sample were analyzed by Western blotting with anti-His, anti-TatB, or anti-FLAG antibodies as indicated. DADE-P coproducing C-terminally FLAG-tagged SufI with its native signal peptide alongside TatB and nontagged TatC (lane annotated “No Histag”) was used as a negative control. Equivalent volumes of sample were loaded in each lane. WT, wild type.

A previous study identified suppressors in the TatB component that could also restore transport activity to substitutions in the TatC twin-arginine binding site. It was shown that substrate precursors could be copurified with wild-type TatBC complexes but did not copurify when the signal peptide binding site was mutated, even in the presence of the TatB suppressors. Thus, it was concluded that the TatB suppressors did not detectably restore binding of signal peptides to the TatBC complex (27). To determine whether the suppressors that we identified here that increase signal peptide hydrophobicity could restore binding to TatBC complexes harboring the TatC^F94D^ substitution, we coproduced FLAG-tagged variants of SufI with these suppressors alongside TatB and His-tagged TatC. After purification of TatBC complexes from digitonin-treated cell lysates, we assessed the level of copurifying SufI by immunoblotting. As shown in Fig. 6, the single substitutions S12L, G13L, A15L, or G19L in the SufI signal peptide did not detectably affect interaction of SufI with wild-type TatBC, since qualitatively similar levels of FLAG-tagged SufI were seen to copurify with TatBC-His. When the F94D substitution was present in His-tagged TatC, no SufI-FLAG was copurified with the variant TatBC-His complex (Fig. 6), even though SufI was clearly detected in the cell lysate (Fig. S6). However, when the S12L, G13L, A15L, or G19L substitution was introduced into SufI, it could now be detected in the fractions containing purified TatBC^F94D^-His. Qualitatively, it appeared that more SufI-FLAG copurified with TatBC^F94D^-His when a stronger suppressing substitution (S12L or G13L) was present than when a weaker suppressing substitution (A15 or G19) was present, suggesting that an increased degree of binding was associated with better suppression. Taken together, these observations indicate that the SufI h-region suppressors restore some degree of signal peptide binding to the TatBC^F94D^ complex.

10.1128/mBio.00909-17.6 FIG S6  Detection of SufI-FLAG variants in input samples prior to Ni affinity purification. Cells of strain DADE-P coproducing C-terminally FLAG-tagged SufI with its native signal peptide (WT SufI) or harboring the indicated leucine substitutions, alongside TatB and C-terminally His-tagged TatC or TatC^F94D^, were lysed and incubated with digitonin. Thirty microliters of the digitonin-solubilized fraction was mixed with an equal volume of 2× Laemmli buffer, and 10 μl of each sample was subjected to SDS-PAGE (12% acrylamide) followed by Western blotting with anti-FLAG antibody. Download FIG S6, PDF file, 0.1 MB. Copyright © 2017 Huang and Palmer. 2017 Huang and Palmer This content is distributed under the terms of the Creative Commons Attribution 4.0 International license.

### Highly hydrophobic signal peptides are compatible with the Tat pathway.

It has previously been reported that the relatively low hydrophobicity of Tat signal peptides partially prevents their routing to the Sec pathway (6). It is not clear, however, whether low h-region hydrophobicity is a mechanistic requirement for engagement with Tat. To explore this in more detail, we investigated the effect of further increasing the hydrophobicity of the SufI signal peptide on transport of the SufIss-AmiA fusion. To this end, we introduced an S12L/G13L double substitution and two quadruple substitutions, S12L/G13L/I14L/A15L and C17L/A18LG19L/A20L, into the SufI signal sequence. These substitutions markedly increase the signal sequence hydrophobicity score, bringing it into the range of the Sec signal sequences of OmpA and DsbA (Table 1).

**FIG 7:**
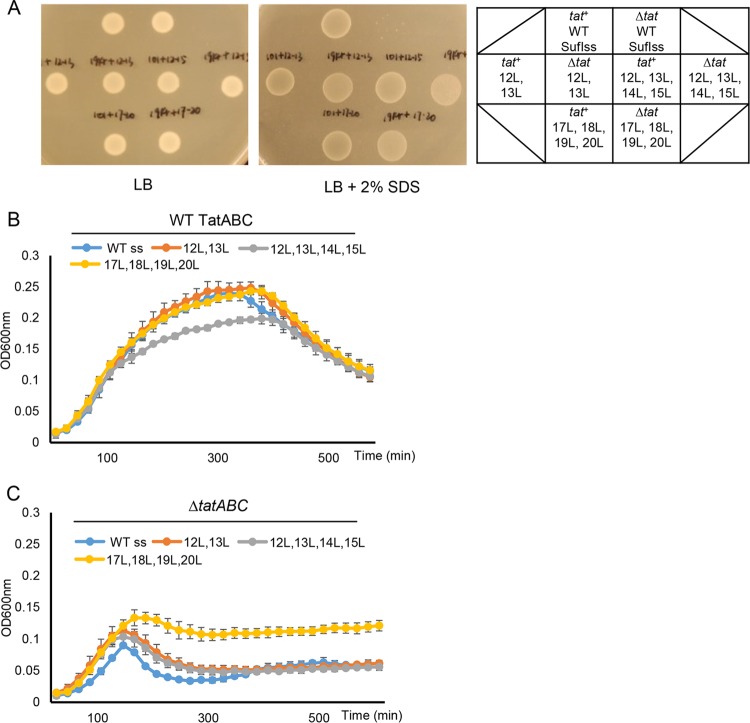
Multiple leucine substitutions in SufI signal peptide h-region partially reroute AmiA to the Sec pathway. Overnight cultures of strain MC4100 Δ*amiA* Δ*amiC* Δ*tatABC* harboring either pTH19kr (Δ*tat*) or pTAT101 producing wild-type (WT) TatABC (*tat*^+^) along with pSUSufIss-mAmiA producing the indicated substitutions in the SufI signal peptide were subcultured at a 1:100 dilution. (A) Cultures were grown for a further 3 h at 37°C, pelleted, and resuspended to an OD_600_ of 0.1, and 8 μl of sample was spotted onto LB agar or LB agar containing 2% SDS. Plates were incubated at 37°C for 16 h. (B and C) Alternatively, cultures were supplemented with 0.5% SDS (final concentration) and grown at 37°C without shaking. The optical density at 600 nm was monitored every 20 min using a plate reader. Error bars are +standard deviations (*n* = 3 biological replicates).

Figure 7A shows that each of these SufIss-AmiA fusion proteins was able to support growth on solid medium in the presence of SDS, although growth was also seen in a strain lacking the Tat pathway, indicating that there is some export of these more hydrophobic SufIss-AmiA fusion proteins by Sec. These findings were confirmed by monitoring growth of these strains in liquid culture (Fig. 7B and C). However, it is clear that in the absence of a functional Tat system, growth in the presence of SDS was much poorer than when the Tat system was present. This observation suggests that there must be some recognition of these hydrophobic signal peptide variants by the Tat pathway. To confirm this, we coproduced FLAG-tagged S12L/G13L/I14L/A15L and C17L/A18LG19L/A20L SufI variants alongside TatBC-His. When His-tagged TatC was purified from digitonin-solubilized cell lysates, each of these hydrophobic SufI-FLAG variants was copurified (Fig. 6), indicating that they retained the ability to interact with the TatBC complex. Taken together, these results show that highly hydrophobic signal peptides are mechanistically compatible with the Tat pathway.

### The OmpA and DsbA signal peptides functionally interact with the Tat pathway.

Our results collectively show that the hallmark twin arginines of Tat signal peptides are not a mechanistic requirement for Tat-dependent transport and that a single arginine or twin lysines in the n-region are compatible with the Tat pathway if compensatory mutations are introduced that increase the hydrophobicity of the signal peptide. Interestingly, many Sec-dependent signal peptides share these parameters (Table S1), raising the possibility that bona fide Sec signal peptides may be able to interact with the Tat pathway. To explore this, we selected two well-studied Sec signal peptides—those of OmpA, which is a posttranslational Sec substrate, and of DsbA, which directs cotranslational translocation (43, 44) (Table 1)—and fused their signal peptides to the mature portion of AmiA. We also made two additional constructs where we introduced a “Sec-avoidance” lysine residue into the signal peptide c-regions, to reduce interaction with the Sec pathway (8).

10.1128/mBio.00909-17.7 TABLE S1  *E. coli* proteins with known or predicted signal peptides. Protein sequences encoded by *E. coli* MG1655 were retrieved from UniProt (http://www.uniprot.org) and analyzed for the presence of a signal peptide using the SignalP 4.1 server (http://www.cbs.dtu.dk/services/SignalP). The n-regions were analyzed manually; twin arginines are colored red, twin lysines are colored green, and Lys-Arg or Arg-Lys is shown in blue. Pairings of Arg-Asn, Arg-Asp, Arg-Gln, Arg-Glu, and Arg-His (which, from this study, can be suppressed by increased signal peptide hydrophobicity) are shown in orange. For those signal peptides harboring any of these dipeptides in their n-regions, the approximate location of the h-region is shown underlined. The start of the h-region was defined by Phobius (http://phobius.sbc.su.se/), and the end of the h-region and the cleavage site was defined by SignalP. Pairings within five residues of the start of the h-region were considered likely to interact with the Tat pathway. Those marked as N? have one of the indicated pairings 5 or more residues away from the h-region and are considered unlikely to interact with the Tat pathway. Known or probable Tat substrates are shown in yellow highlight; EfeO is predicted to be a Tat substrate, but this has not been demonstrated experimentally. Download TABLE S1, DOCX file, 0.04 MB. Copyright © 2017 Huang and Palmer. 2017 Huang and Palmer This content is distributed under the terms of the Creative Commons Attribution 4.0 International license.

**FIG 8:**
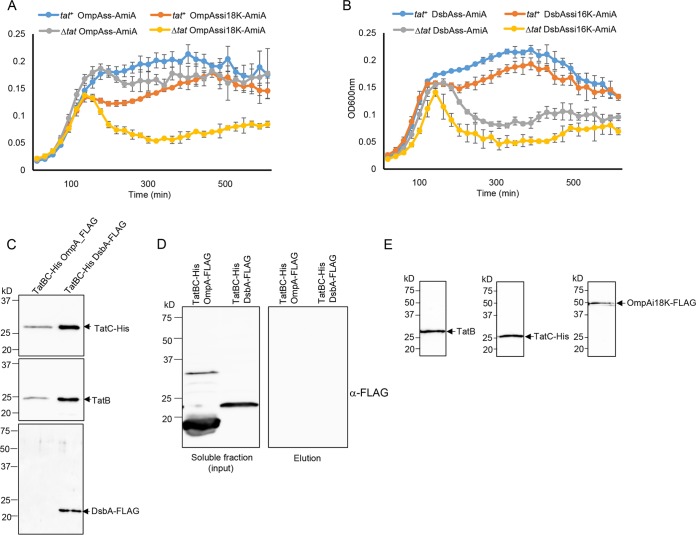
The OmpA and DsbA signal peptides are able to functionally engage with the Tat machinery. (A and B) Overnight cultures of strain MC4100 Δ*amiA* Δ*amiC* Δ*tatABC* harboring either pTH19kr (Δ*tat*) or pTAT101 producing wild-type TatABC (*tat*^+^) along with a plasmid encoding the indicated signal peptide fusion to AmiA were subcultured at a 1:100 dilution, supplemented with 0.5% SDS (final concentration), and grown at 37°C without shaking. The optical density at 600 nm (*y* axis of both panels) was monitored every 20 min using a plate reader. Error bars are +standard deviations (*n* = 3 biological replicates). (C) Cells of strain DADE coproducing TatB, C-terminally His-tagged TatC, and either C-terminally FLAG-tagged DsbA or OmpA, as indicated, were lysed and incubated with digitonin, and His-tagged TatC was isolated using Ni-charged beads. Following elution of bound TatC-His, equivalent volumes of the eluate from each sample were analyzed by Western blotting with anti-His, anti-TatB, or anti-FLAG antibodies. (D) An aliquot cell lysate prior to digitonin treatment from the experiment shown in panel C was ultracentrifuged to remove the cell membranes. A small amount of the supernatant was retained as the input fraction, and the remainder was incubated with Ni-charged beads. The beads were washed three times with wash buffer, and aliquots of the input and eluate samples were analyzed by Western blotting using an anti-FLAG antibody. (E) Cells of DADE-P coproducing C-terminally FLAG-tagged SufI fused to the OmpA signal peptide harboring a lysine insertion after codon 17, alongside TatB and C-terminally His-tagged TatC, were treated as described in the legend to Fig. 6, and equivalent volumes of the elution fraction were analyzed by Western blotting with anti-His, anti-TatB, or anti-FLAG antibodies as indicated.

Figure 8A shows that there is Sec-dependent transport of AmiA mediated by the OmpA signal peptide, as there is strong growth of the Δ*tat* strain producing OmpAss-AmiA in the presence of SDS. Introduction of a lysine at position 18 of the OmpA signal peptide clearly reduces interaction with the Sec pathway, as growth of the Δ*tat* strain producing this variant is significantly reduced. However, there is good growth of the *tat*^+^ strain producing this variant fusion protein, indicating that some of this fusion must be interacting with the Tat pathway. Similarly, Fig. 8B shows that there is some low-level growth of the Δ*tat* strain producing DsbAss-AmiA in SDS-containing medium, which is reduced by inclusion of a Sec-avoidance lysine in the c-region of the DsbA signal peptide. In contrast, the *tat*^+^ strain harboring either of these fusion proteins shows markedly stronger growth in the presence of SDS, indicating that the DsbA signal peptide is productively engaging with the Tat machinery.

To confirm that these signal peptides are able to interact with Tat, we coproduced C-terminally FLAG-tagged variants of full-length OmpA or full-length DsbA alongside TatBC-His. When His-tagged TatC was purified from digitonin-solubilized cell lysates, DsbA-FLAG, which migrated very close to the expected mass of 24.1 kDa, was seen to copurify (Fig. 8C). This copurification was clearly dependent on the presence of the Tat proteins, since when membranes were removed by an ultracentrifugation step, the cytoplasmic form of DsbA-FLAG was no longer isolated by Ni-affinity purification (Fig. 8D). We conclude that FLAG-tagged but otherwise native DsbA can interact with TatBC. In contrast, we were not able to detect copurification of FLAG-tagged OmpA with TatBC under these conditions (Fig. 8C). However, in these experiments we noted that OmpA-FLAG migrated at a lower mass than the predicted size of the tagged protein (38.2 kDa) or of folded OmpA (which migrates at an estimated mass of approximately 30 kDa [45]), raising the possibility that it may have been subjected to proteolysis. We therefore took a second approach to assessing whether the OmpA signal peptide could interact with TatBC by fusing the OmpA signal peptide variant containing the K18 insertion to the N terminus of mature SufI and coproducing it with TatBC-His. Figure 8E indicates that this fusion protein could indeed be copurified alongside TatB and His-tagged TatC, indicating that the OmpA signal peptide is able to interact with the TatBC complex.

## DISCUSSION

In this study, we have sought to identify SufI signal sequence variants that restore Tat transport activity in the presence of substitutions that inactivate the twin-arginine recognition site on TatC. Our results have shown that an increase in signal peptide hydrophobicity can overcome two different inactivating substitutions, TatC^F94D^ and TatC^E103K^, and that these suppressors act to restore detectable binding of the SufI signal sequence to the variant TatBC^F94D^ complex. We further showed that the same hydrophobic substitutions can act in *cis* to compensate for a range of inactivating substitutions at the SufI signal peptide twin-arginine motif. These results demonstrate that neither the consecutive arginines of the signal peptide nor the conserved recognition site on the cytoplasmic surface of TatC is mechanistically essential for operation of the Tat pathway and that they can be bypassed if the signal peptide hydrophobicity is increased. Taken together, our findings indicate that the signal peptide features that can facilitate interaction with the Tat pathway are remarkably similar to those that facilitate interaction with Sec, namely, the presence of at least one basic charge in the n-region and a relatively hydrophobic h-region. Indeed, we show that even a highly hydrophobic signal peptide that naturally directs its passenger into the cotranslocational Sec pathway can functionally engage with the Tat system.

If the Tat pathway can interact with hydrophobic signal peptides lacking the twin-arginine motif, why then do almost all Tat substrates that have been identified contain paired arginine residues and only moderately hydrophobic h-regions? In prokaryotes and plant chloroplasts, the Tat system always coexists with the Sec pathway. In bacteria, ribosome-associated signal recognition particle (SRP) and cytosolic or ribosome-bound SecA capture Sec substrates at an early stage of biogenesis, at least partially through interaction with their signal sequences (46, 47). Signal sequence hydrophobicity is a key sorting feature for Sec substrates; highly hydrophobic signals generally interact with SRP, whereas those with lower hydrophobicity bind to SecA (46). Photo-cross-linking and/or genetic studies have indicated that Tat signal peptides interact with ribosomally bound trigger factor and with general cytoplasmic chaperones, including DnaK (48–51), but no cross-links to SecA have been reported, and *in vitro* analysis indicates that Tat signal peptides do not productively engage with SecA to the same extent as a Sec signal peptide (52). It is therefore likely that Tat signal peptides evolved lower hydrophobicity to avoid the targeting pathways that feed into the Sec translocon and that the paired arginines and the twin-arginine binding site are necessary features to strengthen recognition of these weakly hydrophobic signal peptides by the Tat machinery. In this context, it is interesting that although paired arginines in the signal peptide n-region are compatible with the Sec pathway, this pairing is relatively rare in Sec signal peptides, at least in *E. coli*, being found in only five of the 244 probable Sec signal peptides listed in Table S1 in the supplemental material (compared with 53 that have paired lysines). If lysine and arginine are equivalent in the amino-terminal region of a Sec signal peptide, as implied by kinetic analysis (53), this might suggest that there is selection pressure against the presence of paired arginines in Sec signals.

The presence of one or more positively charged amino acids in the c-region of Tat signal peptides is a further feature that has no mechanistic requirement for Tat translocation but leads to rejection of these signal sequences by the Sec pathway (6, 8, 9). C-terminal positive charges may act at a late stage during Sec translocation when the signal peptide is already engaged with the Sec translocon, and Sec avoidance motifs are particularly abundant in membrane proteins that require the dual action of the Sec and Tat pathways for their assembly (7, 54). Here, the Tat-dependent signal sequence (which is internal to the protein and follows a series of Sec-dependent transmembrane domains) has several c-region positive charges that result in abortive interaction with the Sec pathway, freeing up the sequence to be recognized by Tat (7). Taken together, it is clear that there is strong selective pressure, particularly at the level of Tat signal peptides, to refine features that minimize mistargeting to the Sec pathway.

Our findings show that signal peptides with either twin lysines or an unpaired arginine, coupled with a moderately hydrophobic h-region, can functionally interact with the Tat pathway. Inspection of all of the signal peptides present at the N termini of *E. coli* MG1655 proteins identified using SignalP 4.1 (http://www.cbs.dtu.dk/services/SignalP [55]) indicates that some 44% of Sec signal peptides contain either KR, RK, KK, RD, RE, RH, RN, or RQ adjacent to their h-regions (Table S1) and therefore potentially have the capability of engaging with the Tat pathway. Whether any of these would ever target to the Tat pathway *in vivo* is not clear, since presumably under standard conditions *E. coli* synthesizes sufficient targeting factors to ensure that Sec substrates are efficiently channeled into the Sec pathway. This is particularly difficult to envisage for substrates that are sequestered by SRP at the ribosome and cotranslationally targeted to the Sec translocon. However, there may be exceptional situations when SRP is out-titrated, resulting in SRP-free ribosomes, and it would presumably be under these circumstances where a substrate might escape targeting to Sec and engage with the Tat pathway. Interestingly, it should be noted that in *Bacillus subtilis*, hyperproduction of a normally Sec-dependent lipase results in overflow into the Tat pathway (56), raising the possibility that transient rerouting of substrates to the Tat pathway may occur on occasions where cells undergo secretion stress.

## MATERIALS AND METHODS

### Strain and plasmid construction.

Strains used in this study are MC4100 derivatives (57). Strain MC4100 Δ*amiA* Δ*amiC* Δ*tatABC* (F^−^ Δ*lacU169 araD139 rpsL150 relA1 ptsF rbs flbB5301* Δ*amiA* Δ*amiC* Δ*tatABC*) was used for signal peptide library screening and for SDS growth tests with signal peptide-AmiA fusion proteins (27). Strain DADE (as MC4100, Δ*tatABCD* Δ*tatE* [58]) was used for SufI transport assays, and DADE-P [as DADE, *pcnB1 zad*-*981*::Tn*10*d (Kan^r^) (59)] was used for copurification experiments.

All plasmids used and constructed in this study are given in Table S2 in the supplemental material (65, 66). Point mutations in plasmids were introduced by QuikChange site-directed mutagenesis (Stratagene) using the primers listed in Table S3. Plasmid pTAT101 was used for low-level production of TatA, TatB, and TatC (37). Plasmid pSUSufIss-mAmiA was used to produce SufIss-AmiA, where the SufI signal peptide is fused to the mature portion of AmiA (27). Plasmid pQE80-SufIhis was used to produce His-tagged SufI (27).

10.1128/mBio.00909-17.8 TABLE S2  Plasmids used and constructed in this study. Download TABLE S2, DOCX file, 0.02 MB. Copyright © 2017 Huang and Palmer. 2017 Huang and Palmer This content is distributed under the terms of the Creative Commons Attribution 4.0 International license.

10.1128/mBio.00909-17.9 TABLE S3  Oligonucleotides used in this study. Download TABLE S3, DOCX file, 0.02 MB. Copyright © 2017 Huang and Palmer. 2017 Huang and Palmer This content is distributed under the terms of the Creative Commons Attribution 4.0 International license.

Plasmids pSUDsbAss-mAmiA, pSUDsbAssi16K-mAmiA (with an additional lysine codon inserted after codon 15 of the DbsA signal peptide), pSUOmpAss-mAmiA, and pSUOmpAssi18K-mAmiA (with an additional lysine codon inserted after codon 17 of the OmpA signal peptide) were constructed according to the method in reference 27. Briefly, DNA fragments encoding DsbAss, DsbAssi16K, OmpAss, and OmpAssi18K were amplified by PCR using MC4100 genomic DNA as the template, using primer pairs DsbAss-FE/DsbAss-R, DsbAss-FE/DsbAss16inK-R, OmpA-FE/OmpAss-R, and OmpA-FE/OmpA18inK-R, respectively. DNA fragments encoding the corresponding mature domain of AmiA were amplified by PCR using MC4100 genomic DNA as the template with primer pair OmpA-mAmiA-F/amiA-mRX, or DsbA-mAmiA-F/amiA-mRX. The DNA fragments encoding the signal peptides and the mature domain of AmiA were fused by overlap extension PCR, giving DNA fragments DsbAss-mAmiA, DsbAssi16K-mAmiA, OmpAss-mAmiA, and OmpAi18Kss-mAmiA, which were finally cloned into the pSU18 vector following digestion with EcoRI and XbaI.

Plasmid pFAT75BC-SufIFLAG was modified from pFAT75ΔA-SufIhis (18) via QuikChange using primers FAT75SufIFLAG-1/FAT75SufIFLAG-2. Plasmid pFATBChis-SufIFLAG was modified from pFAT75BC-SufIFLAG via QuikChange using primers FAT75TatChis-1/FAT75TatChis-2. Plasmid pFATBChis-OmpAssi18KSufIFLAG has the SufI signal peptide coding region substituted for DNA encoding OmpAssi18K and was constructed using a restriction enzyme-free cloning method according to the method in reference 60. Briefly, a DNA fragment covering OmpAssi18K was PCR amplified using pSUOmpAssi18K-mAmiA as the template, with primer pair FATHF-OmpA-F/FATHF-OmpA18K-R. The resultant DNA fragment was used as a primer to amplify the whole pFATBChis-SufIFLAG plasmid using a PCR program of 95°C for 2 min followed by 15 cycles of 95°C for 30 s, 48°C for 1.5 min, and 68°C for 15 min and a final extension at 68°C for 10 min. The PCR product was subjected to DpnI digestion and introduced into *E. coli* JM109 competent cells by transformation. The resultant plasmid was verified by DNA sequencing. Plasmids pQEBChis-OmpAFLAG and pQEBChis-DsbAFLAG were used for coproduction of TatB, His-tagged TatC, and FLAG-tagged OmpA or DsbA, respectively, and were constructed as follows. A DNA fragment encoding TatB and His-tagged TatC was amplified using pFATBChis-SufIFLAG as the template with primer pair QEF/FAT75TatChis-2 and was ligated, via an ApaI restriction site, to a DNA fragment encoding FLAG-tagged OmpA (which was amplified using MC4100 genomic DNA as the template with primer pair FATHF-OmpA-F/OmpAFLAG-SR) or FLAG-tagged DsbA (amplified similarly using primer pair FATHF-OmpA-F/DsbAFLAG-SR). The ligated fragment was enriched by using the ligation mixture as a template in a PCR with primer pair QEF/OmpAFLAG-SR or QEF/DsbAAFLAG-SR, respectively. The amplified fragment was gel purified, digested with EcoRI and SalI, and cloned into similarly digested pQE80. Constructs were verified by DNA sequencing.

### Mutant library construction and screening.

To construct a random library of substitutions at codon 8 of the SufI signal peptide, site-directed mutagenesis was carried out via QuikChange using a pair of random primers, SufIF8X1 and SufIF8X2 (Table S3), and pSUSufIss-mAmiA as the template. The PCR product was subsequently introduced into XL1-Gold ultracompetent cells (Agilent). Transformants were scraped from plates, resuspended in LB, pooled, and cultured overnight, after which plasmid DNA was isolated and taken as the F8X random library. The library contained approximately 5,000 clones, and random sequencing of eight of them revealed mutations of the TTC codon to CTT, ATC GGT, GTG, GGT, CAA, AGA, and GGG.

The signal peptide mutagenesis library in plasmid pSUSufIss-mAmiA was constructed as described previously (27). Briefly, an error-containing DNA fragment covering the *sufI* signal sequence was amplified by error-prone PCR using primers SufIF and SufIR and pSUSufIss-mAmiA as the template. This fragment was used as a megaprimer to amplify the whole pSUSufIss-mAmiA plasmid. The amplified plasmid was introduced into XL1-Gold ultracompetent cells following nick repair using T4 polynucleotide kinase and T4 ligase. Transformants were scraped from plates, resuspended in LB, pooled, and used to inoculate fresh LB to an initial optical density at 600 nm (OD_600_) of 0.2. Cells were grown at 37°C until OD_600_ reached 2, after which plasmid DNA was prepared and taken as the signal peptide mutagenesis library.

For library screening, plasmid pTAT101 harboring the *tatC* point substitution of interest (along with wild-type *tatAB*) was introduced into MC4100 Δ*amiA* Δ*amiC* Δ*tatABC*. Subsequently, the mutant library was introduced, and cells were plated onto LB agar containing 2% SDS. Plasmids were isolated from colonies growing on this selective medium, and mutations were identified by sequencing.

### Protein methods.

Copurification of TatBC-substrate complexes was carried out as described previously (27). Briefly, an overnight culture of DADE-P harboring plasmid pFATBChis-SufIFLAG or its derivatives was subcultured in LB supplemented with 0.5% glycerol and appropriate antibiotics for 2.5 h at 37°C with shaking. Following supplementation with 0.4 mM isopropyl-β-d-galactopyranoside (IPTG), cells were incubated overnight at 30°C. The following morning, cells were harvested, resuspended in 200 μl of 2× lysis buffer (100 mM NaH_2_PO_4_, pH 8.0, 600 mM NaCl, 40 mM imidazole, 50 mg lysozyme, DNase I, and protease inhibitor), and mixed gently at room temperature for 1 h. Cells were then frozen at −80°C for 1 h and thawed at room temperature. An equal volume of 2.5% digitonin was added to the cells, and the samples were solubilized for 1 h at 4°C. The insoluble material was pelleted by centrifugation at 4°C. A 30-μl amount of the supernatant was mixed with 2× Laemmli buffer, which was taken as the input sample, and the remaining supernatant was mixed with 50 μl wash buffer-equilibrated nickel beads (Profinity IMAC Ni-charged resin; Bio-Rad,; catalog number 156-0131) for 1 h. The nickel beads were pelleted, washed three times with 1 ml wash buffer (50 mM NaH_2_PO_4_, pH 8.0, 300 mM NaCl, 40 mM imidazole, 0.03% digitonin), and then mixed with 100 μl elution buffer (50 mM NaH_2_PO_4_, pH 8.0, 300 mM NaCl, 700 mM imidazole, 0.03% digitonin). The beads were incubated for 10 min with shaking and then pelleted. The supernatant (elution fraction) was taken and mixed with an equal volume of 2× Laemmli buffer, and 20 μl of the sample was subjected to SDS-PAGE followed by Western blotting with anti-His (anti-6×His tag antibody [GT359] [horseradish peroxidase {HRP}]; Abcam, Inc.; catalog number ab184607), anti-TatB (61), or anti-FLAG antibodies (monoclonal anti-FLAG M2 antibody produced in mouse; Sigma; catalog number F1804). Secondary antibody was goat anti-rabbit IgG (HRP conjugate; Bio-Rad; catalog number 170-6515) or goat anti-mouse IgG (HRP conjugate; Bio-Rad; catalog number 1706516).

Subcellular fractionation was carried out as described previously (27). All experiments were carried out with at least three biological replicates, and representative results are shown. Briefly, overnight cultures of strain DADE harboring pTAT101 or the cognate empty vector pTH19kr along with pQE80-SufIhis or its derivatives were subcultured at 1:50 in LB supplemented with 1 mM IPTG and grown at 37°C until OD_600_ reached 1. Where the RR-KK substitution was present in the SufI, no IPTG was used (as for unknown reasons this substitution results in high-level expression of SufI even in the absence of IPTG). For whole-cell samples, cells were pelleted from 5 ml of the culture, resuspended in 250 μl resuspension buffer (50 mM Tris-HCl, pH 7.6, 2 mM EDTA), and lysed by sonicating for 15 s. The cell lysate was mixed with an equal volume of 2× Laemmli buffer and boiled at 95°C for 10 min. For preparation of periplasm, cells were pelleted from 20 ml of the culture and resuspended in 500 μl fractionation buffer (20 mM Tris-HCl, pH 7.6, 2 mM EDTA, 20% [wt/vol] sucrose). Freshly made lysozyme (0.6 mg/ml) was added, and the cells were incubated at room temperature for 20 min. The cells were then pelleted by centrifugation, and the supernatant was taken and mixed with an equal volume of 2× Laemmli buffer. Aliquots (20 μl) of the whole-cell or periplasmic fraction samples were separated by SDS-PAGE (10% acrylamide) followed by Western blotting with anti-His antibody or anti-His and anti-RNA polymerase β-subunit mixed antibodies.

### Prediction of Sec signal peptides.

All of the protein sequences encoded by *E. coli* MG1655 were analyzed using the SignalP 4.1 server (http://www.cbs.dtu.dk/services/SignalP) (55) with parameters “Gram-negative bacteria” and “input sequence do not include TM regions” selected. Inner membrane proteins were removed manually from the output Sec substrate candidates.
