# Supplementary figures and images for: Signal Peptide Hydrophobicity Modulates Interaction with the Twin-Arginine Translocase
Source: mBio. 2017 Aug 1;8(4):e00909-17. doi: 10.1128/mBio.00909-17 (PMC5539426; doi:10.1128/mBio.00909-17)

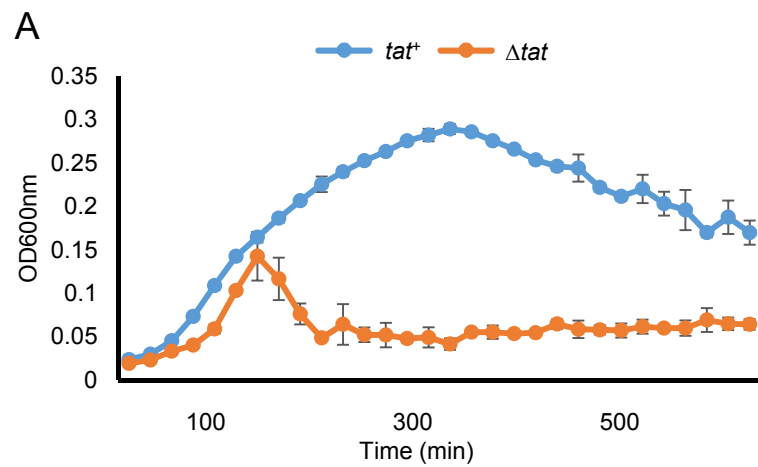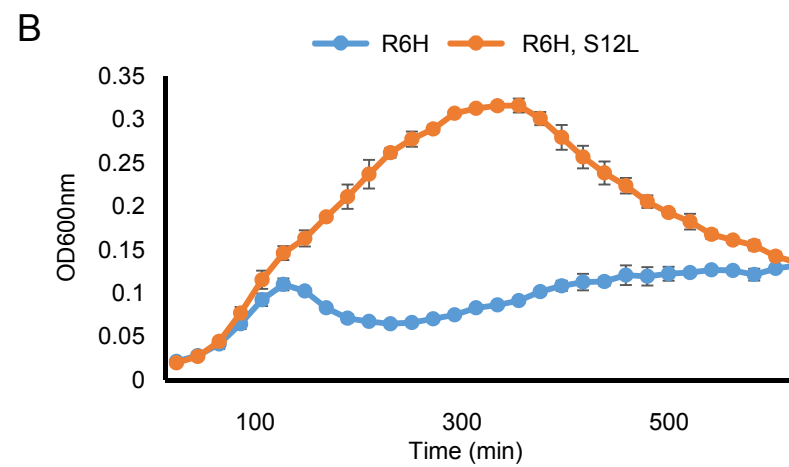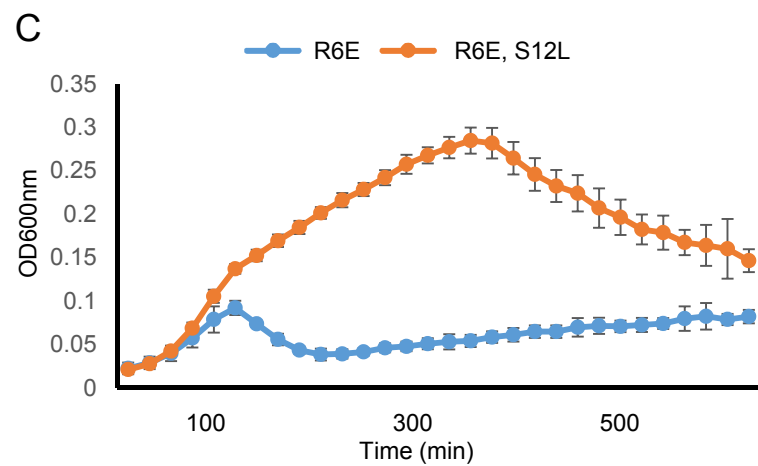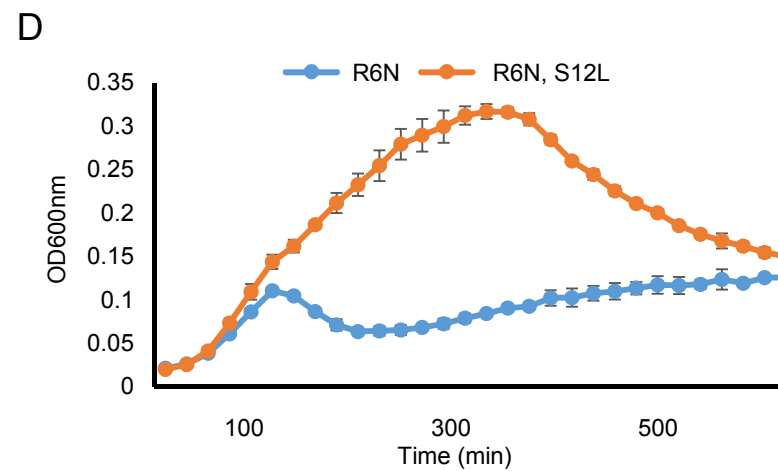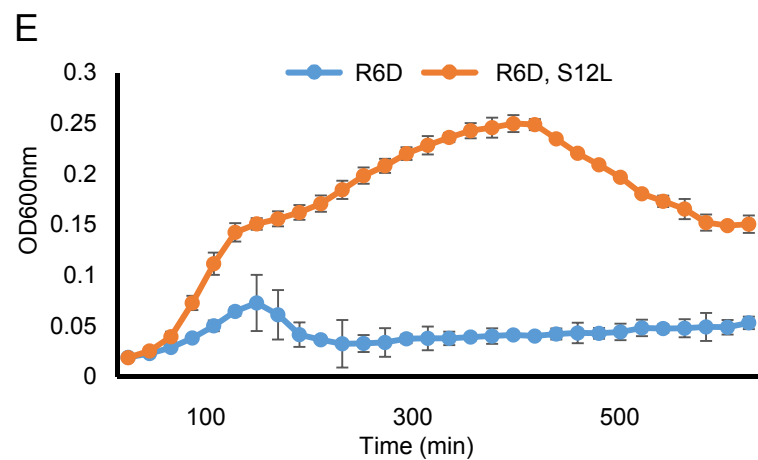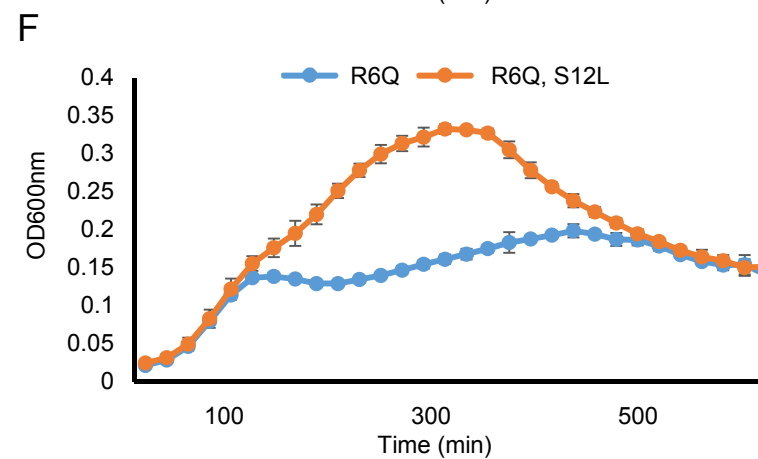

G

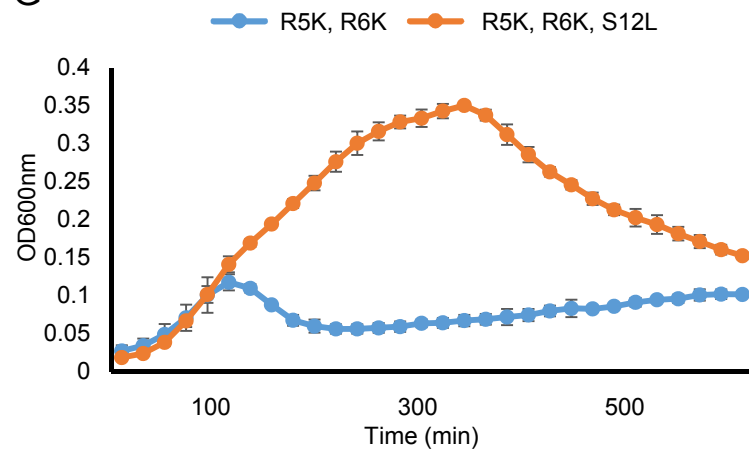

H

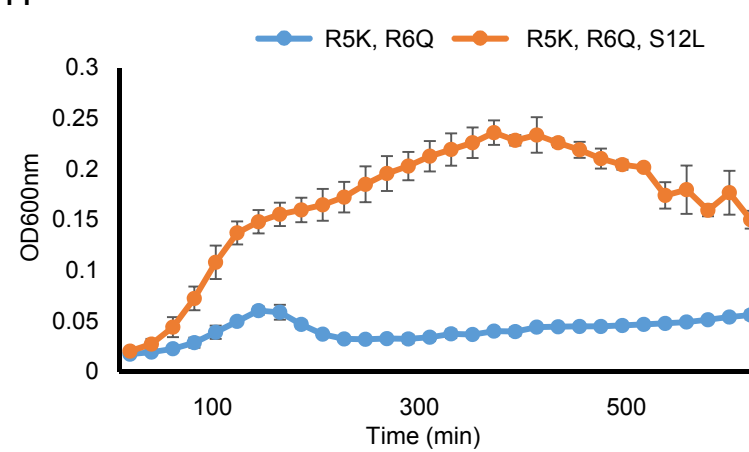

I

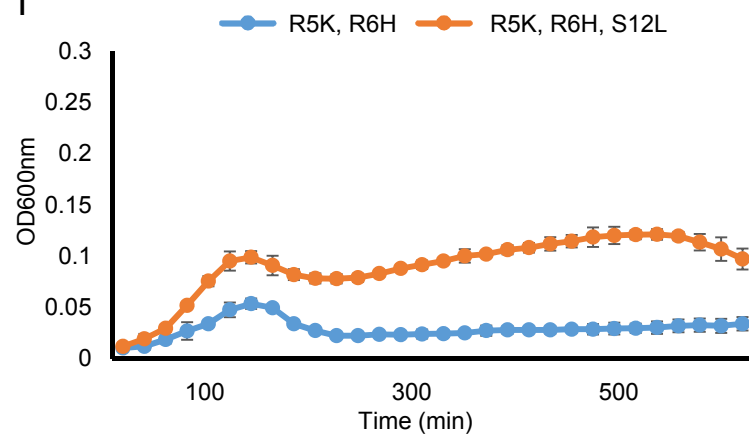

J

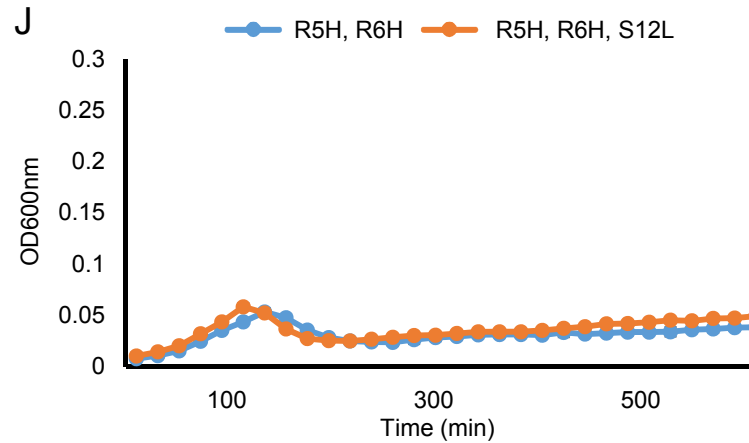

Supplement: FIG S1 [file mbo004173403sf1.pdf]

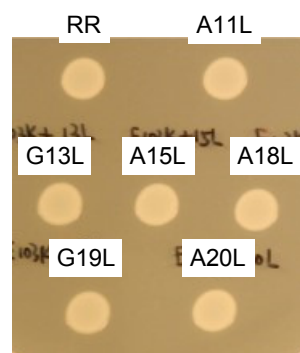

LB

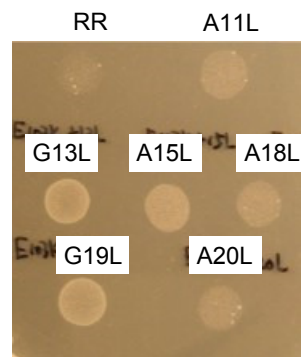

LB + 2% SDS

Supplement: FIG S3 [file mbo004173403sf3.pdf]

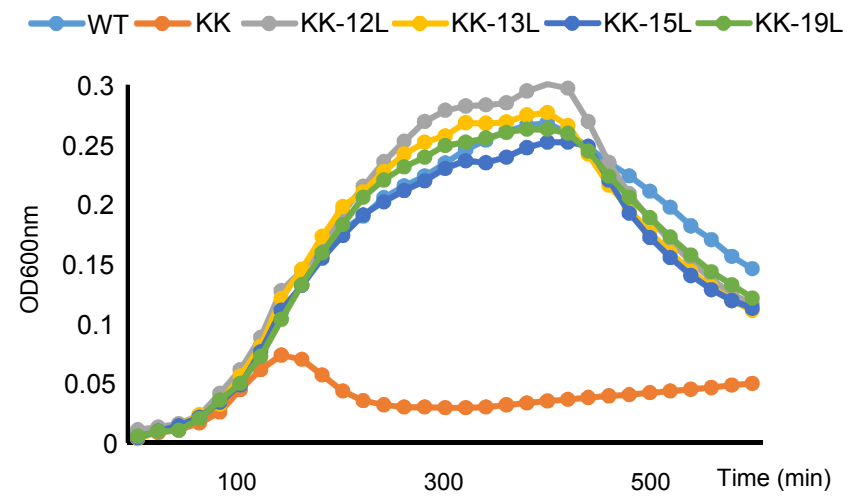

Supplement: FIG S4 [file mbo004173403sf4.pdf]

**A**

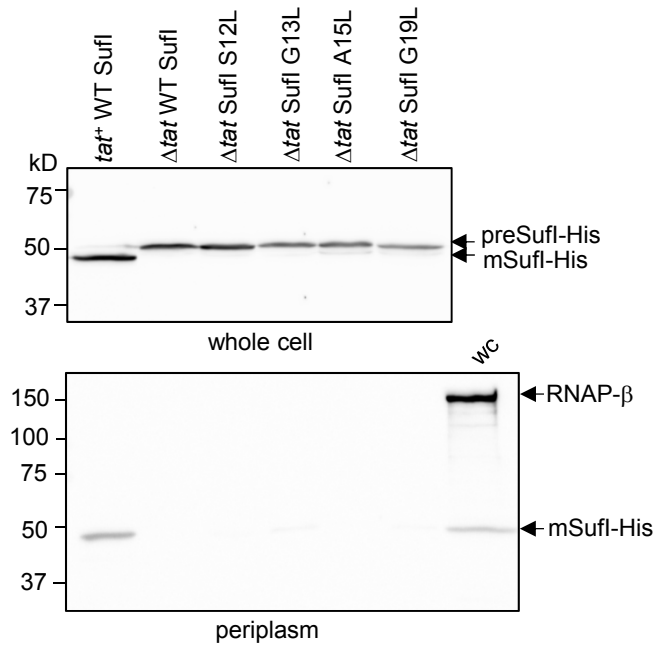

**B**

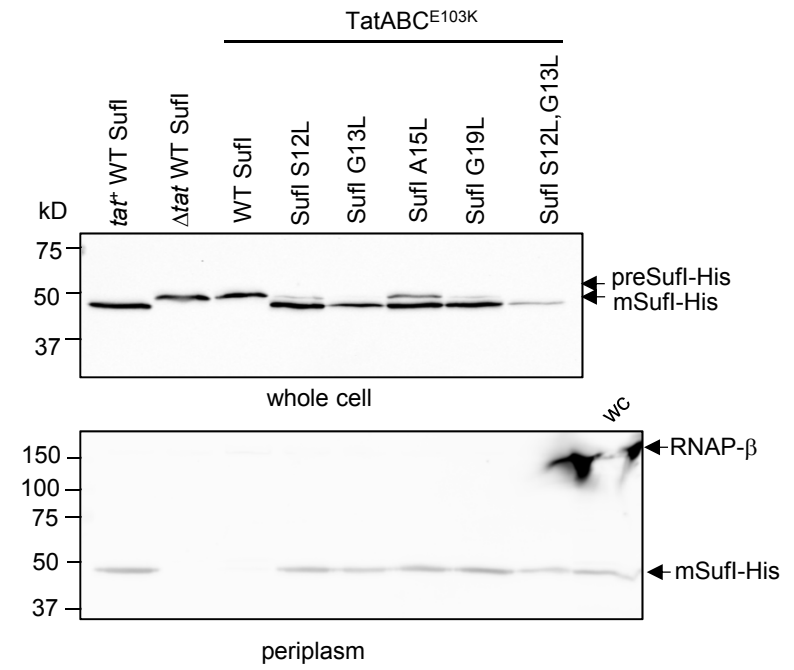

Supplement: FIG S5 [file mbo004173403sf5.pdf]

Input

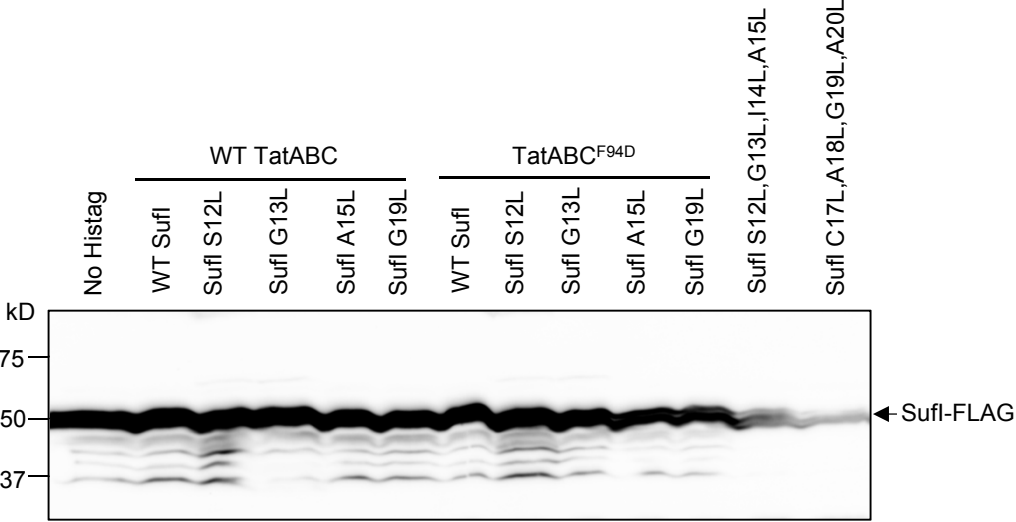

Supplement: FIG S6 [file mbo004173403sf6.pdf]
