# Supplementary material for: Signal Peptide Hydrophobicity Modulates Interaction with the Twin-Arginine Translocase
Source: mBio. 2017 Aug 1;8(4):e00909-17. doi: 10.1128/mBio.00909-17 (PMC5539426; doi:10.1128/mBio.00909-17)

A

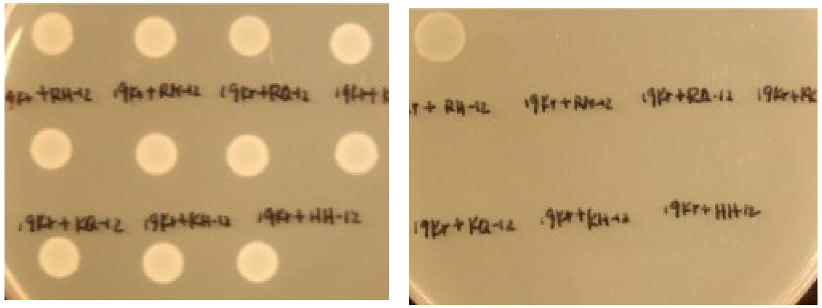

LB   LB + 2% SDS

tat⁺	Δ*tat*	R6D S12L	R6E S12L
R6H S12L	R6N S12L	R6Q S12L	R5K R6K S12L
R5K R6Q S12L	R5K R6H S12L	R5H R6H S12L	

B

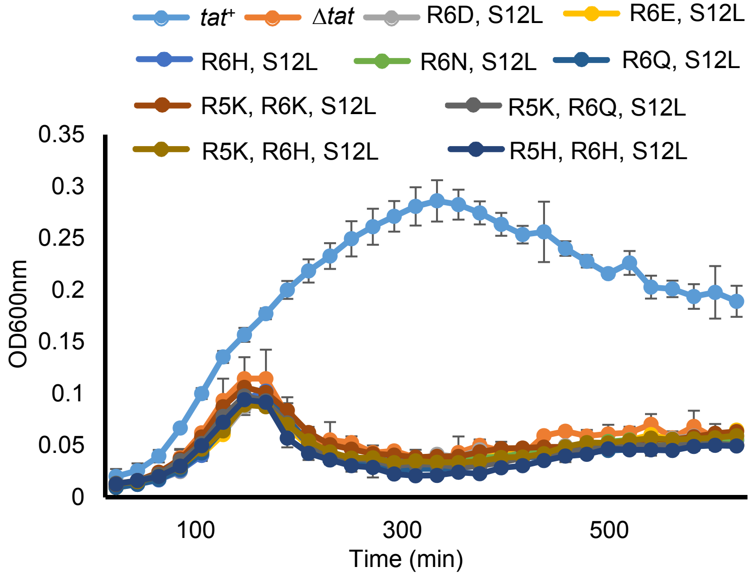

Supplement: FIG S2 [file mbo004173403sf2.pdf]
