# Supplementary material for: Signal Peptide Hydrophobicity Modulates Interaction with the Twin-Arginine Translocase
Source: mBio. 2017 Aug 1;8(4):e00909-17. doi: 10.1128/mBio.00909-17 (PMC5539426; doi:10.1128/mBio.00909-17)
Supplement: TABLE S1 [file mbo004173403st1.docx]

| **Protein ID** | **Gene name** | **Uniprot Description** | **Signal sequence** | **Subcellular location** | **Likely Tat interaction?** |
| --- | --- | --- | --- | --- | --- |
| >NP_414546.1 | *yaaX* | Uncharacterized protein YaaX | MKKMQSIVLALSLVLVAPMAAQA | Periplasm | Y |
| >NP_414917.2 | *phoA* | Alkaline phosphatase | MKQSTIALALLPLLFTPVTKA | [Periplasm](http://www.uniprot.org/locations/SL-0200) |  |
| >NP_414554.1 | *yaaI* | UPF0412 protein YaaI | MKSVFTISASLAISLMLCCTAQA | Periplasm |  |
| >NP_414678.1 | *yadK* | Uncharacterized fimbrial-like protein YadK | MHPTQRKLMKRIILFLSLLFCIACPAIA | Fimbrium | Y |
| >NP_414682.1 | *yadV* | Probable fimbrial chaperone YadV | MFFNTKHTTALCFVTCMAFSSSSIA | Periplasm |  |
| >NP_414683.1 | *yadN* | Uncharacterized fimbrial-like protein YadN | MSKKLGFALSGLMLAMVAGTASA | Fimbrium | Y |
| >NP_414692.1 | *fhuA* | Ferrichrome-iron receptor | MARSKTAQPKHSLRKIAVVVATAVSGMSVYAQA | OM | Y |
| >NP_414694.1 | *fhuD* | Iron(3+)-hydroxamate-binding protein FhuD | MSGLPLISRRRLLTAMALSPLLWQMNTAHA | Periplasm | Tat substrate |
| >NP_414700.1 | *btuF* | Vitamin B12-binding protein | MAKSLFRALVALSFLAPLWLNAA | Periplasm |  |
| >NP_414703.1 | *degP* | Periplasmic serine endoprotease DegP | MKKTTLALSALALSLGLALSPLSATA | Periplasm | Y |
| >NP_414719.1 | *bamA* | Outer membrane protein assembly factor BamA | MAMKKLLIASLLFSSATVYG | OM | Y |
| >NP_414720.1 | *skp* | Chaperone protein Skp | MKKWLLAAGLGLALATSAQA | Periplasm | Y |
| >NP_414734.1 | *nlpE* | Lipoprotein NlpE | MVKKAIVTAMAVISLFTLMGC | OM | Y |
| >NP_414735.4 | *yaeF* | Probable endopeptidase YaeF | MDKPKAYCRLFLPSFLLLSAC | Membrane |  |
| >NP_414752.1 | *yafT* | Uncharacterized lipoprotein YafT | MNSKKLCCICVLFSLLAGC | Membrane | Y |
| >NP_414755.1 | *ivy* | Inhibitor of vertebrate lysozyme | MGRISSGGMMFKAITTVAALVIATSAMA | Periplasm |  |
| >NP_414762.1 | *yafL* | Probable endopeptidase YafL | MSLPSIPSFVLSGLLLICLPFSSFA | Periplasm |  |
| >NP_414776.1 | *phoE* | Outer membrane pore protein E | MKKSTLALVVMGIVASASVQA | OM | Y |
| >NP_414784.1 | *ykfB* | Uncharacterized protein YkfB | MTILSLSRFMLAGVLLASFNASA | Periplasm |  |
| >NP_414785.4 | *yafY* | Lipoprotein YafY | MKRKTLPLLALVATTLFLIAC | IM | Y |
| >NP_414825.1 | *ecpC* | Probable outer membrane usher protein EcpC | MPLRRFSPGLKAQFAFGMVFLFVQPDASA | Periplasm | N? |
| >NP_414827.1 | *ecpA* | Common pilus major fimbrillin subunit EcpA | MKKKVLAIALVTVFTGMGVAQA | Fimbrium | Y |
| >NP_414858.1 | *yahJ* | Uncharacterized protein YahJ | MKESNSRREFLSQSGKMVTAAALFGTSVPLAHA | Periplasm | Tat substrate |
| >NP_414863.1 | *yahO* | Uncharacterized protein YahO | MKIISKMLVGALALAVTNVYA | Periplasm |  |
| >NP_414899.2 | *tauA* | Taurine-binding periplasmic protein | MAISSRNTLLAALAFIAFQAQA | Periplasm | Y |
| >NP_414910.1 | *ampH* | D-alanyl-D-alanine-carboxypeptidase/endopeptidase AmpH | MKRSLLFSAVLCAASLTSVHA | Periplasm | Y |
| >NP_414918.4 | *psiF* | Phosphate starvation-inducible protein PsiF | MKITLLVTLLFGLVFLTTVGA | Periplasm |  |
| >NP_414946.4 | *yajI* | Uncharacterized lipoprotein YajI | MNTNVFRLLLLGSLFSLSAC | Membrane |  |
| >NP_414968.4 | *yajG* | Uncharacterized lipoprotein YajG | MFKKILFPLVALFMLAGC | Membrane | Y |
| >NP_414976.1 | *ybaV* | Uncharacterized protein YbaV | MKHGIKALLITLSLACAGMSHSALA | Periplasm |  |
| >NP_414987.3 | *ybaY* | Uncharacterized lipoprotein YbaY | MKLVHMASGLAVAIALA | OM |  |
| >NP_414998.1 | *mscK* | Mechanosensitive channel MscK | MTMFQYYKRSRHFVFSAFIAFVFVLLC | IM | Y |
| >NP_415013.1 | *ushA* | Protein UshA | MKLLQRGVALALLTTFTLASETALA | Periplasm |  |
| >NP_415027.1 | *tesA* | Acyl-CoA thioesterase 1 | MMNFNNVFRWHLPFLFLVLLTFRAAA | Periplasm |  |
| >NP_415031.1 | *ybbC* | Uncharacterized protein YbbC | MKYSSIFSMLSFFILFAC | Membrane |  |
| >NP_415063.4 | *sfmA* | Uncharacterized fimbrial-like protein SfmA | MKLRFISSALAAALFAATGSYA | Fimbrium |  |
| >NP_415064.1 | *sfmC* | Probable fimbrial chaperone SfmC | MMTKIKLLMLIIFYLIISASAHA | Periplasm |  |
| >NP_415077.1 | *ybcL* | UPF0098 protein YbcL | MKTLIVSTVLAFITFSAQAAA | Periplasm |  |
| >NP_415100.1 | *nfrA* | Bacteriophage adsorption protein A | MKENNLNRVIGWSGLLLTSLLSTSALA | OM |  |
| >NP_415105.1 | *cusF* | Cation efflux system protein CusF | MKKALQVAMFSLFTVIGFNAQA | Periplasm | Y |
| >NP_415116.1 | *fepA* | Ferrienterobactin receptor | MNKKIHSLALLVNLGIYGVAQA | OM | Y |
| >NP_415124.1 | *fepB* | Ferrienterobactin-binding periplasmic protein | MRLAPLYRNALLLTGLLLSGIA | Periplasm | Y |
| >NP_415137.2 | *dsbG* | Thiol:disulfide interchange protein DsbG | MLKKILLLALLPAIAFA | Periplasm | Y |
| >NP_415144.1 | *rna* | Ribonuclease I | MKAFWRNAALLAVSLLPFSSANA | Periplasm & Cytoplasm? | Y |
| >NP_415155.1 | *pagP* | Lipid A palmitoyltransferase PagP | MNVSKYVAIFSFVFIQLISVGKVFANA | OM |  |
| >NP_415166.1 | *rlpA* | Endolytic peptidoglycan transglycosylase RlpA | MRKQWLGICIAAGMLAAC | Membrane | Y |
| >NP_415188.1 | *gltI* | Glutamate/aspartate import solute-binding protein | MQLRKPATAILALALSAGLAQA | Periplasm | Y |
| >NP_415207.1 | *chiP* | Chitoporin | MRTFSGKRSTLALAIAGVTAMSGFMA | OM | Y |
| >NP_415208.1 | *chiQ* | Uncharacterized lipoprotein ChiQ | MKKLILIAIMASGLVAC | Membrane | Y |
| >NP_415215.1 | *ybfP* | Uncharacterized lipoprotein YbfP | MKTNRSLVVIVSLITATLLLTAC | Membrane |  |
| >NP_415228.1 | *ybfA* | Uncharacterized protein YbfA | MELYREYPAWLIFLRRTYAVA | Periplasm | Y |
| >NP_415244.4 | *ybgO* | Uncharacterized protein YbgO | MSAGKGLLLVICLLFLPLKSAMA | Fimbrium |  |
| >NP_415245.1 | *ybgP* | Uncharacterized fimbrial chaperone YbgP | MTFIKGLPLMLLTISLGCNA | Periplasm |  |
| >NP_415246.2 | *ybgQ* | Uncharacterized outer membrane usher protein YbgQ | MNIYRLSFVSCLVMAMPCAMA | OM |  |
| >NP_415268.1 | *tolB* | Protein TolB | MKQALRVAFGFLILWASVLHA | Periplasm |  |
| >NP_415269.1 | *pal* | Peptidoglycan-associated lipoprotein | MQLNKVLKGLMIALPVMAIAAC | OM |  |
| >NP_415270.1 | *cpoB* | Cell division coordinator CpoB | MSSNFRHQLLSLSLLVGIAAPWAAFA | Periplasm | Y |
| >NP_415274.1 | *ybgS* | Uncharacterized protein YbgS | MKMTKLATLFLTATLSLASGAALA | Periplasm |  |
| >NP_415284.1 | *modA* | Molybdate-binding periplasmic protein | MARKWLNLFAGAALSFAVAGNA | Periplasm | Y |
| >NP_415293.1 | *ybhC* | Putative acyl-CoA thioester hydrolase YbhC | MNTFSVSRLALALAFGVTLTAC | OM |  |
| >NP_415323.1 | *ybiJ* | Uncharacterized protein YbiJ | MKTINTVVAAMALSTLSFGVFA | Periplasm |  |
| >NP_415326.1 | *fiu* | Catecholate siderophore receptor Fiu | MENNRNFPARQFHSLTFFAGLCIGITPVAQAALA | OM | Y |
| >NP_415327.2 | *mcbA* | Uncharacterized protein McbA | MKKLLIATVLSGISLTAYA | Periplasm | Y |
| >NP_415332.1 | *glnH* | Glutamine-binding periplasmic protein | MKSVLKVSLAALTLAFAVSSHA | Periplasm |  |
| >NP_415335.1 | *ompX* | Outer membrane protein X | MKKIACLSALAAVLAFTAGTSVA | OM | Y |
| >NP_415340.1 | *ybiS* | Probable L,D-transpeptidase YbiS | MNMKLKTLFAAAFAVVGFCSTASA | Periplasm |  |
| >NP_415351.1 | *gsiB* | Glutathione-binding protein GsiB | MARAVHRSGLVALGIATALMASCAFA | Periplasm |  |
| >NP_415355.1 | *yliF* | Putative lipoprotein YliF | MSRINKFVLTVSLLIFIMISAVAC | Membrane |  |
| >NP_415358.1 | *yliI* | Aldose sugar dehydrogenase YliI | MHRQSFFLVPLICLSSALWA | Periplasm | Y |
| >NP_415360.1 | *dacC* | D-alanyl-D-alanine carboxypeptidase DacC | MTQYSSLLRGLAAGSAFLFLFAPTAFA | Periplasm |  |
| >NP_415364.1 | *ybjH* | Uncharacterized protein YbjH | MIMKNCLLLGALLMGFTGVAMA | Periplasm |  |
| >NP_415375.1 | *potF* | Putrescine-binding periplasmic protein | MTALNKKWLSGLVAGALMAVSVGTLA | Periplasm | Y |
| >NP_415381.1 | *artJ* | ABC transporter arginine-binding protein 1 | MKKLVLAALLASFTFGASA | Periplasm | Y |
| >NP_415384.1 | *artI* | Putative ABC transporter arginine-binding protein 2 | MKKVLIAALIAGFSLSATA | Periplasm | Y |
| >NP_415386.1 | *ybjP* | Uncharacterized lipoprotein YbjP | MRYSKLTMLIPCALLLSAC | Membrane |  |
| >NP_415411.2 | *lolA* | Outer-membrane lipoprotein carrier protein | MKKIAITCALLSSLVASSVWA | Periplasm | Y |
| >NP_415446.1 | *ycbK* | Uncharacterized protein YcbK | MDKFDANRRKLLALGGVALGAAILPTPAFA | Periplasm | Tat substrate |
| >NP_415449.1 | *ompF* | Outer membrane protein F | MMKRNILAVIVPALLVAGTANA | OM | Y |
| >NP_415456.4 | *ssuA* | Putative aliphatic sulfonates-binding protein | MRNIIKLALAGLLSVSTFAVA | Periplasm | Y |
| >NP_415458.2 | *elfA* | Fimbrial subunit ElfA | MKKSVLTAFITVVCATSSVMA | Fimbrium | Y |
| >NP_415459.1 | *elfD* | Probable fimbrial chaperone protein ElfD | MKTCITKGIVTVSLTAILLSCSSAWA | Periplasm |  |
| >NP_415460.1 | *elfC* | Probable outer membrane usher protein ElfC | MYRTHRQHSLLSSGGVPSFIGGLVVFVSAAFNAQA | OM | Y |
| >NP_415464.4 | *ycbF* | Uncharacterized fimbrial chaperone YcbF | MTNTWNRLALLIFAVLSLLVAGELQA | Periplasm |  |
| >NP_415472.2 | *ymbA* | Uncharacterized lipoprotein YmbA | MKKWLVTIAALWLAGC | Membrane | Y |
| >NP_415477.1 | *ompA* | Outer membrane protein A | MKKTAIAIAVALAGFATVAQA | OM | Y |
| >NP_415484.1 | *yccT* | UPF0319 protein YccT | MKTGIVTTLIALCLPVSVFA | Periplasm |  |
| >NP_415491.1 | *hyaA* | Hydrogenase-1 small chain | MNNEETFYQAMRRQGVTRRSFLKYCSLAATSLGLGAGMAPKIAWA | IM | Tat substrate |
| >NP_415500.1 | *appA* | Periplasmic AppA protein | MKAILIPFLSLLIPLTPQSAFA | Periplasm |  |
| >NP_415503.1 | *gfcE* | Putative polysaccharide export protein GfcE | MKKNIFKFSVLTLAVLSLTAC | OM | Y |
| >NP_415504.1 | *gfcD* | Uncharacterized lipoprotein GfcD | MKKNSYLLSCLAIAVSSAC | Membrane | Y |
| >NP_415505.1 | *gfcC* | Uncharacterized protein GfcC | MNKLQSYFIASVLYVMTPHAFA | Periplasm |  |
| >NP_415506.1 | *gfcB* | Uncharacterized lipoprotein GfcB | MRPLILSIFALFLAGC | Membrane |  |
| >NP_415522.1 | *agp* | Glucose-1-phosphatase | MNKTLIAAAVAGIVLLASNAQA | Periplasm |  |
| >NP_415537.1 | *efeO* | Iron uptake system component EfeO | MTINFRRNALQLSVAALFSSAFMANA | Periplasm | Tat substrate? |
| >NP_415538.1 | *efeB* | Deferrochelatase/peroxidase EfeB | MQYKDENGVNEPSRRRLLKVIGALALAGSCPVAHA | Periplasm | Tat substrate |
| >NP_415542.1 | *pgaB* | Poly-beta-1,6-N-acetyl-D-glucosamine N-deacetylase | MLRNGNKYLLMLVSIIMLTAC | OM | Y |
| >NP_415559.1 | *csgB* | Minor curlin subunit | MKNKLLFMMLTILGAPGIAAA | Fimbrium |  |
| >NP_415560.1 | *csgA* | Major curlin subunit | MKLLKVAAIAAIVFSGSALA | Fimbrium |  |
| >NP_415562.1 | *ymdA* | Uncharacterized protein YmdA | MFRPFLNSLMLGSLFFPFIAIA | Periplasm |  |
| >NP_415566.1 | *mdoG* | Glucans biosynthesis protein G | MMKMRWLSAAVMLTLYTSSSWA | Periplasm |  |
| >NP_415574.1 | *yceI* | Protein YceI | MKKSLLGLTFASLMFSAGSAVA | Periplasm | Y |
| >NP_415590.1 | *flgA* | Flagella basal body P-ring formation protein FlgA | MLIIKRSVAIIAILFSPLSTA | Periplasm | Y |
| >NP_415598.3 | *flgI* | Flagellar P-ring protein | MIKFLSALILLLVTTAAQA | Periplasm |  |
| >NP_415620.1 | *fhuE* | FhuE receptor | MLSTQFNRDNQYQAITKPSLLAGCIALALLPSAAFA | OM | N? |
| >NP_415630.1 | *bhsA* | Multiple stress resistance protein BhsA | MKNVKTLIAAAILSSMSFASFA | OM |  |
| >NP_415631.1 | *ycfS* | Probable L,D-transpeptidase YcfS | MMIKTRFSRWLTFFTFAAAVALA | Periplasm |  |
| >NP_415641.1 | *potD* | Spermidine/putrescine-binding periplasmic protein | MKKWSRHLLAAGALALGMSAAHA | Periplasm | Y |
| >NP_415696.1 | *pliG* | Inhibitor of g-type lysozyme | MKIKSIRKAVLLLALLTSTSFA | Periplasm | Y |
| >NP_415715.1 | *treA* | Periplasmic trehalase | MKSPAPSRPQKMALIPACIFLCFAALSVQA | Periplasm |  |
| >NP_415738.2 | *ychO* | Uncharacterized protein YchO | MSRFVPRIIPFYLLLLVAGGTANA | Periplasm |  |
| >NP_415759.1 | *oppA* | Periplasmic oligopeptide-binding protein | MTNITKRSLVAAGVLAALMAGNVALA | Periplasm | Y |
| >NP_415772.1 | *ompW* | Outer membrane protein W | MKKLTVAALAVTTLLSGSAFA | OM | Y |
| >NP_415799.1 | *osmB* | Osmotically-inducible lipoprotein B | MFVTSKKMTAAVLAITLAMSLSAC | Membrane | Y |
| >NP_415810.1 | *sapA* | Probable ABC transporter periplasmic-binding protein SapA | MRQVLSSLLVIAGLVSGQAIA | Periplasm | Y |
| >NP_415826.1 | *ycjN* | Putative ABC transporter periplasmic-binding protein YcjN | MIKSKIVLLSALVSCALIS | Periplasm |  |
| >NP_415835.1 | *ompG* | Outer membrane protein G | MKKLLPCTALVMCAGMACAQA | OM | Y |
| >NP_415895.1 | *ompN* | Outer membrane protein N | MKSKVLALLIPALLAAGAAHA | OM |  |
| >NP_415936.1 | *ydcA* | Uncharacterized protein YdcA | MKKLALILFMGTLVSFYADA | Periplasm | Y |
| >NP_415941.5 | *mdoD* | Glucans biosynthesis protein D | MDRRRFIKGSMAMAAVCGTSGIASLFSQAAFA | Periplasm | Tat substrate |
| >NP_415948.1 | *ydcL* | Uncharacterized lipoprotein YdcL | MRTTSFAKVAALCGLLALSGC | Membrane |  |
| >NP_415953.1 | *yncJ* | Uncharacterized protein YncJ | MFTKALSVVLLTCALFSGQLMA | Periplasm |  |
| >NP_415957.1 | *ydcS* | Putative ABC transporter periplasmic-binding protein YdcS | MSKTFARSSLCALSMTIMTAHA | Periplasm |  |
| >NP_415969.1 | *yncE* | Uncharacterized protein YncE | MHLRHLFSSRLRGSLLLGSLLVVSSFSTQA | Periplasm | N? |
| >NP_415991.1 | *fdnG* | Formate dehydrogenase, nitrate-inducible, major subunit | MDVSRRQFFKICAGGMAGTTVAALGFAPKQALA | Periplasm | Tat substrate |
| >NP_416004.1 | *ddpA* | Probable D,D-dipeptide-binding periplasmic protein DdpA | MKRSISFRPTLLALVLATNFPVAHA | Periplasm | N? |
| >NP_416008.1 | *yddW* | UPF0748 lipoprotein YddW | MDICSRNKKLTIRRPAILVALALLLCSC | Membrane | Y |
| >NP_416015.2 | *ydeN* | Uncharacterized sulfatase YdeN | MKSALKKSVVSTSISLILASGMAAFAAHA | Periplasm | Y |
| >NP_416054.1 | *ydeI* | Uncharacterized protein YdeI | MKFQAIVLASFLVMPYALA | Periplasm |  |
| >NP_416100.1 | *ynfB* | UPF0482 protein YnfB | MKITLSKRIGLLAILLPCALALSTTVHA | Periplasm | Y |
| >NP_416103.2 | *ynfD* | Uncharacterized protein YnfD | MKLSTCCAALLLALASPAVLA | Periplasm |  |
| >NP_416114.2 | *asr* | Acid shock protein | MKKVLALVVAAAMGLSSAAFA | Periplasm | Y |
| >NP_416121.1 | *ydgH* | Protein YdgH | MKLKNTLLASALLSAMAFSVNA | Periplasm |  |
| >NP_416132.2 | *uidC* | Membrane-associated protein UidC | MRKIVAMAVICLTAASGLTSAYA | OM? | Y |
| >NP_416163.1 | *sodC* | Superoxide dismutase [Cu-Zn] | MKRFSLAILALVVATGAQA | Periplasm | Y |
| >NP_416172.1 | *mepH* | Murein DD-endopeptidase MepH | MARINRISITLCALLFTTLPLTPMAHA | Periplasm |  |
| >NP_416192.1 | *lpp* | Major outer membrane lipoprotein Lpp | MKATKLVLGAVILGSTLLAGC | OM |  |
| >NP_416193.1 | *ynhG* | Probable L,D-transpeptidase YnhG | MKRASLLTLTLIGAFSAIQAAWA | Periplasm | Y |
| >NP_416223.1 | *nlpC* | Probable endopeptidase NlpC | MRFCLILITALLLAGC | Membrane |  |
| >NP_416236.1 | *ydiY* | Uncharacterized protein YdiY | MKLLKTVPAIVMLAGGMFASLNAAA | OM |  |
| >NP_416257.1 | *spy* | Periplasmic chaperone Spy | MRKLTALFVASTLALGAANLAHA | Periplasm | Y |
| >NP_416274.1 | *ynjH* | Uncharacterized protein YnjH | MSRALFAVVLAFPLIALA | Periplasm |  |
| >NP_416296.1 | *mipA* | MltA-interacting protein | MTKLKLLALGVLIATSAGVAHA | OM |  |
| >NP_416355.1 | *yobA* | Protein YobA | MASTARSLRYALAILTTSLVTPSVWAHA | Periplasm |  |
| >NP_416371.4 | *znuA* | High-affinity zinc uptake system protein ZnuA | MLHKKTLLFAALSAALWGGA | Periplasm | Y |
| >NP_416414.1 | *araF* | L-arabinose-binding periplasmic protein | MHKFTKALAAIGLAAVMSQSAMA | Periplasm |  |
| >NP_416438.1 | *yedD* | Uncharacterized lipoprotein YedD | MKKLAIAGALLLLAGC | Membrane | Y |
| >NP_416482.1 | *zinT* | Metal-binding protein ZinT | MAIRLYKLAVALGVFIVSAPAFS | Periplasm |  |
| >NP_416547.1 | *wcaM* | Colanic acid biosynthesis protein WcaM | MPFKKLSRRTFLTASSALAFLHTPFARA | Periplasm | Tat substrate |
| >NP_416578.2 | *mdtA* | Multidrug resistance protein MdtA | MKGSYKSRWVIVIVVVIAAIA | Periplasm |  |
| >NP_416610.4 | *rcnB* | Nickel/cobalt homeostasis protein RcnB | MTIKNKMLLGALLLVTSAAWAAPA | Periplasm |  |
| >NP_416612.1 | *yehB* | Outer membrane usher protein YehB | MLRMTPLASAIVALLLGIEAYA | OM |  |
| >NP_416613.1 | *yehC* | Probable fimbrial chaperone YehC | MAAIPWRPFNLRGIKMKGLLSLLIFSMVLPAHA | Periplasm |  |
| >NP_416627.2 | *yehR* | Uncharacterized lipoprotein YehR | MKAFNKLFSLVVASVLVFSLAGC | Membrane |  |
| >NP_416636.1 | *bglX* | Periplasmic beta-glucosidase | MKWLCSVGIAVSLALQPALA | Periplasm |  |
| >NP_416638.4 | *pbpG* | D-alanyl-D-alanine endopeptidase | MPKFRVSLFSLALMLAVPFAPQAVA | Periplasm |  |
| >NP_416655.1 | *mglB* | D-galactose-binding periplasmic protein | MNKKVLTLSAVMASMLFGAA | Periplasm | Y |
| >NP_416682.4 | *yejA* | Uncharacterized protein YejA | MIVRILLLFIALFTFGVQAQA | Periplasm |  |
| >NP_416698.1 | *ccmH* | Cytochrome c-type biogenesis protein CcmH | MRFLLGVLMLMISGSALA | Periplasm |  |
| >NP_416710.1 | *napA* | Periplasmic nitrate reductase | MKLSRRSFMKANAVAAAAAAAGLSVPGVARA | Periplasm | Tat substrate |
| >NP_416713.1 | *eco* | Ecotin | MKTILPAVLFAAFATTSAWA | Periplasm |  |
| >NP_416719.1 | *ompC* | Outer membrane protein C | MKVKVLSLLVPALLVAGAANA | OM |  |
| >NP_416742.1 | *glpQ* | Glycerophosphodiester phosphodiesterase, periplasmic | MKLTLKNLSMAIMMSTIVMGSSAMA | Periplasm |  |
| >NP_416812.1 | *hisJ* | Histidine-binding periplasmic protein | MKKLVLSLSLVLAFSSATA | Periplasm | Y |
| >NP_416835.2 | *yfcO* | Uncharacterized protein YfcO | MKILRWLFALVMLIATTEAMA | OM |  |
| >NP_416837.1 | *yfcQ* | Uncharacterized fimbrial-like protein YfcQ | MRKTFLTLLCVSSAIAHA | Fimbrium | Y |
| >NP_416838.1 | *yfcR* | Uncharacterized fimbrial-like protein YfcR | MTGGVMSQKFVVGAGLLVCSVCSLSAMA | Fimbrium |  |
| >NP_416839.1 | *yfcS* | Probable fimbrial chaperone YfcS | MSDLLCSAKLGAMTLALLLSATSLSALA | Periplasm |  |
| >NP_416891.1 | *ypeC* | Uncharacterized protein YpeC | MFRSLFLAAALMAFTPLAANA | Periplasm |  |
| >NP_416930.1 | *amiA* | N-acetylmuramoyl-L-alanine amidase AmiA | MSTFKPLKTLTSRRQVLKAGLAALTLSGMSQAIA | Periplasm | Tat substrate |
| >NP_416944.1 | *yffR* | Uncharacterized protein YffR | MKVLGNILWWAFVGFMAYA | Periplasm |  |
| >NP_416989.1 | *bepA* | Beta-barrel assembly-enhancing protease | MFRQLKKNLVATLIAAMTIGQVAPAFA | Periplasm | Y |
| >NP_417000.1 | *yfgH* | Uncharacterized lipoprotein YfgH | MMKFKKCLLPVAMLASFTLAGC | OM | Y |
| >NP_417007.1 | *bamB* | Outer membrane protein assembly factor BamB | MQLRKLLLPGLLSVTLLSGC | OM | Y |
| >NP_417043.1 | *yphF* | ABC transporter periplasmic-binding protein YphF | MPTKMRTTRNLLLMATLLGSALFARA | Periplasm | Y |
| >NP_417066.1 | *rseB* | Sigma-E factor regulatory protein RseB | MKQLWFAMSLVTGSLLFSANASA | Periplasm |  |
| >NP_417086.1 | *bamD* | Outer membrane protein assembly factor BamD | MTRMKYLVAAATLSLFLAGC | OM |  |
| >NP_417125.1 | *yfjT* | Uncharacterized protein YfjT | MKIRSLSRFVLASTMFASFTASA | Periplasm |  |
| >NP_417289.2 | *ygdI* | Uncharacterized lipoprotein YgdI | MKKTAAIISACMLTFALSAC | Membrane | Y |
| >NP_417294.4 | *amiC* | N-acetylmuramoyl-L-alanine amidase AmiC | MSGSNTAISRRRLLQGAGAMWLLSVSQVSLA | Periplasm | Tat substrate |
| >NP_417369.1 | *dsbC* | Thiol:disulfide interchange protein DsbC | MKKGFMLFTLLAAFSGFAQA | Periplasm | Y |
| >NP_417420.1 | *endA* | Endonuclease-1 | MYRYLSIAAVVLSAAFSGPALA | Periplasm |  |
| >NP_417432.1 | *ansB* | L-asparaginase 2 | MEFFKKTALAALVMGFSGAALA | Periplasm | Y |
| >NP_417445.1 | *yghG* | Uncharacterized lipoprotein YghG | MSIKQMPGRVLISLLLSVTGLLSGC | Membrane |  |
| >NP_417470.1 | *hybA* | Hydrogenase-2 operon protein HybA | MNRRNFIKAASCGALLTGALPSVSHAAA | Periplasm | Tat substrate |
| >NP_417471.1 | *hybO* | Hydrogenase-2 small chain | MTGDNTLIHSHGINRRDFMKLCAALAATMGLSSKAAA | Periplasm | Tat substrate |
| >NP_417489.1 | *ftsP/sufI* | Cell division protein FtsP/SufI | MSLSRRQFIQASGIALCAGAVPLKASA | Periplasm | Tat substrate |
| >NP_417492.1 | *ygiS* | Probable deoxycholate-binding periplasmic protein YgiS | MYTRNLLWLVSLVSAAPLYA | Periplasm | Y |
| >NP_417496.1 | *ygiW* | Protein YgiW | MKKFAAVIAVMALCSAPVMA | Periplasm | Y |
| >NP_417520.1 | *yqiI* | Uncharacterized protein YqiI | MRYLLIVITFFMGFSSLPAWA | Fimbrium |  |
| >NP_417551.1 | *ygjK* | Glucosidase YgjK | MKIKTILTPVTCALLISFSAHA | Periplasm |  |
| >NP_417611.1 | *yraH* | Uncharacterized fimbrial-like protein YraH | MNKVTKTAIAGLLALFAGNAAA | Fimbrium |  |
| >NP_417612.1 | *yraI* | Probable fimbrial chaperone YraI | MSKRTFAVILTLLCSFCIGQALA | Periplasm | Y |
| >NP_417614.1 | *yraK* | Uncharacterized fimbrial-like protein YraK | MKRAPLITGLLLISTSCAYA | Fimbrium | Y |
| >NP_417659.1 | *mlaC* | Probable phospholipid-binding protein MlaC | MFKRLMMVALLVIAPLSAATA | Periplasm | Y |
| >NP_417667.1 | *lptA* | Lipopolysaccharide export system protein LptA | MKFKTNKLSLNLVLASSLLAASIPAFA | Periplasm |  |
| >NP_417681.1 | *gltF* | Protein GltF | MFFKKNLTTAAICAALSVAAFSAMA | Periplasm | Y |
| >NP_417683.1 | *yhcD* | Uncharacterized outer membrane usher protein YhcD | MLKKTLLAYTIGFAFSPPANA | OM | Y |
| >NP_417686.1 | *yhcF* | Uncharacterized protein YhcF | MNNVKLLIAGSAFFAMSAQA | Cytoplasm/  Periplasm? |  |
| >NP_417701.1 | *degQ* | Periplasmic pH-dependent serine endoprotease DegQ | MKKQTQLLSALALSVGLTLSASFQAVA | Periplasm | Y |
| >NP_417705.2 | *yhcN* | Uncharacterized protein YhcN | MKIKTTVAALSVLSVLSFGAFA | Periplasm |  |
| >NP_417797.1 | *chiA* | Probable bifunctional chitinase/lysozyme | MKLNIFTKSMIGMGLVCSALPALA | Periplasm |  |
| >NP_417806.1 | *fkpA* | FKBP-type peptidyl-prolyl cis-trans isomerase FkpA | MKSLFKVTLLATTMAVALHAPITFAAEA | Periplasm |  |
| >NP_417822.1 | *ppiA* | Peptidyl-prolyl cis-trans isomerase A | MFKSTLAAMAAVFALSALSPAAMA | Periplasm |  |
| >NP_417904.1 | *ggt* | Gamma-glutamyltranspeptidase | MIKPTFLRRVAIAALLSGSCFSAAA | Periplasm | Y |
| >NP_417910.1 | *ugpB* | sn-glycerol-3-phosphate-binding periplasmic protein UgpB | MKPLHYTASALALGLALMGNAQA | Periplasm |  |
| >NP_417915.1 | *livK* | Leucine-specific-binding protein | MKRNAKTIIAGMIALAISHTAMA | Periplasm | Y |
| >NP_417933.1 | *nikA* | Nickel-binding periplasmic protein | MLSTLRRTLFALLACASFIVHAAA | Periplasm | Y |
| >NP_417963.4 | *slp* | Outer membrane protein slp | MNMTKGALILSLSFLLAAC | OM |  |
| >NP_417966.4 | *hdeB* | Acid stress chaperone HdeB | MNISSLRKAFIFMGAVAALSLVNAQSALA | Periplasm | Y |
| >NP_417984.1 | *yhjJ* | Protein YhjJ | MQGTKIRLLAGGLLMMATAGYVQA | Periplasm |  |
| >NP_418023.1 | *xylF* | D-xylose-binding periplasmic protein | MKIKNILLTLCTSLLLTNVAAHA | Periplasm |  |
| >NP_418028.1 | *malS* | Periplasmic alpha-amylase | MKLAACFLTLLPGFAVA | Periplasm |  |
| >NP_418041.1 | *yiaT* | Putative outer membrane protein YiaT | MLINRNIVALFALPFMASATA | OM | Y |
| >NP_418070.6 | *envC* | Murein hydrolase activator EnvC | MTRAVKPRRFAIRPIIYASVLSAGVLLCAFSAHA | Periplasm | Y |
| >NP_418117.1 | *nlpA* | Lipoprotein 28 | MKLTTHHLRTGAALLLAGILLAGC | IM |  |
| >NP_418184.1 | *pstS* | Phosphate-binding protein PstS | MKVMRTTVATVVAATLSMSAFSVFAEA | Periplasm |  |
| >NP_418207.1 | *rbsB* | Ribose import binding protein RbsB | MNMKKLATLVSAVALSATVSANAMA | Periplasm | Y |
| >NP_418245.1 | *aslA* | Arylsulfatase | MEFSFSPKRLVVAVAAALPLMASA | Periplasm | Y |
| >NP_418297.1 | *dsbA* | Thiol:disulfide interchange protein DsbA | MKKIWLALAGLVLAFSASA | Periplasm | Y |
| >NP_418311.1 | *ompL* | Porin OmpL | MKKINAIILLSSLTSASVFAGA | OM | Y |
| >NP_418355.1 | *yiiQ* | Uncharacterized protein YiiQ | MKPGCTLFFLLCSALTVTTEAHA | Periplasm |  |
| >NP_418372.1 | *yiiX* | Uncharacterized protein YiiX | MKNRLLILSLLVSVPAFA | Periplasm |  |
| >NP_418401.1 | *btuB* | Vitamin B12 transporter BtuB | MIKKASLLTACSVTAFSAWA | OM | Y |
| >NP_418430.2 | *zraP* | Zinc resistance-associated protein | MKRNTKIALVMMALSAMAMGSTSAFA | Periplasm | Y |
| >NP_418450.1 | *yjbE* | Uncharacterized protein YjbE | MKKVLYGIFAISALAATSAWA | Periplasm | Y |
| >NP_418452.1 | *yjbG* | Uncharacterized protein YjbG | MIKQTIVALLLSVGASSVFA | Periplasm |  |
| >NP_418458.1 | *malE* | Maltose-binding periplasmic protein | MKIKTGARILALSALTTMMFSASA | Periplasm |  |
| >NP_418460.1 | *lamB* | Maltoporin | MMITLRKLPLAVAVAAGVMSAQAMA | OM | Y |
| >NP_418461.1 | *malM* | Maltose operon periplasmic protein | MKMNKSLIVLCLSAGLLASA | Periplasm |  |
| >NP_418479.1 | *aphA* | Class B acid phosphatase | MRKITQAISAVCLLFALNSSAVA | Periplasm | Y |
| >NP_418495.2 | *nrfB* | Cytochrome c-type protein NrfB | MSVLRSLLTAGVLASGLLWSLNGITATPAAQA | Periplasm |  |
| >NP_418499.1 | *nrfF* | Formate-dependent nitrite reductase complex subunit NrfF | MNKGLLTLLLLFTCFAHA | Periplasm |  |
| >NP_418502.1 | *yjcO* | Uncharacterized protein YjcO | MKKIIALMLFLTFFAHA | Periplasm | Y |
| >NP_418507.2 | *yjcS* | Putative alkyl/aryl-sulfatase YjcS | MNNSRLFRLSRIVIALTAASGMMVNTANA | Periplasm |  |
| >NP_418512.1 | *alsB* | D-allose-binding periplasmic protein | MNKYLKYFSGTLVGLMLSTSAFA | Periplasm |  |
| >NP_418529.1 | *phnD* | Phosphonates-binding periplasmic protein | MNAKIIASLAFTSMFSLSTLLSPAHA | Periplasm |  |
| >NP_418620.1 | *yjfY* | Uncharacterized protein YjfY | MFSRVLALLAVLLLSANTWA | Periplasm |  |
| >NP_418641.1 | *tamA* | Translocation and assembly module TamA | MRYIRQLCCVSLLCLSGSAVA | OM | Y |
| >NP_418648.1 | *ytfQ* | ABC transporter periplasmic-binding protein YtfQ | MWKRLLIVSAVSAAMSSMALA | Periplasm | Y |
| >NP_418710.4 | *fecB* | Fe(3+) dicitrate-binding periplasmic protein | MLAFIRFLFAGLLLVISHAFA | Periplasm |  |
| >NP_418711.1 | *fecA* | Fe(3+) dicitrate transport protein FecA | MTPLRVFRKTTPLVNTIRLSLLPLAGLSFSAFA | OM | N? |
| >NP_418730.4 | *nanM* | N-acetylneuraminate epimerase | MNKTITALAIMMASFAANA | Periplasm |  |
| >NP_418734.1 | *fimA* | Type-1 fimbrial protein, A chain | MKIKTLAIVVLSALSLSSTAALA | Fimbrium |  |
| >NP_418735.2 | *fimI* | Fimbrin-like protein FimI | MKRKRLFLLASLLPMFALA | Fimbrium | Y |
| >NP_418736.3 | *fimC* | Chaperone protein FimC | MSNKNVNVRKSQEITFCLLAGILMFMAMMVAGRAEA | Periplasm | Y |
| >NP_418737.1 | *fimD* | Outer membrane usher protein FimD | MSYLNLRLYQRNTQCLHIRKHRLAGFFVRLVVACAFAAQAPLSSA | OM | Y |
| >NP_418738.1 | *fimF* | Protein FimF | MRNKPFYLLCAFLWLAVSHALA | Fimbrium | Y |
| >NP_418739.1 | *fimG* | Protein FimG | MKWCKRGYVLAAILALASATIQA | Fimbrium | Y |
| >NP_418793.1 | *osmY* | Osmotically-inducible protein Y | MTMTRLKISKTLLAVMLTSAVATGSAYA | Periplasm |  |
| >NP_418809.4 | *slt* | Soluble lytic murein transglycosylase | MEKAKQVTWRLLAAGVCLLTVSSVARA | Periplasm |  |
| >NP_418814.1 | *creA* | Protein CreA | MKYKHLILSLSLIMLGPLAHA | Periplasm |  |
| >YP_001165332.1 | *yjbT* | Uncharacterized protein YjbT | MKRNLIKVVKMKPYFAALMLSVSVLPAYA | Periplasm | N? |
| >YP_002791241.1 | *rzoQ* | Putative lipoprotein RzoQ | MRNRNLLKFLPGLLICLIVLTSC | Membrane | Y |
| >YP_026195.1 | *yqhG* | Uncharacterized protein YqhG | MKIILLFLAALASFTVHA | Periplasm |  |
| >YP_026223.1 | *livJ* | Leu/Ile/Val-binding protein | MNIKGKALLAGCIALAFSNMALA | Periplasm |  |
| >YP_026240.1 | *yidX* | Uncharacterized protein YidX | MKLNFKGFFKAAGLFPLALMLSGC | Membrane |  |
| >YP_026277.1 | *cpxP* | Periplasmic protein CpxP | MRIVTAAVMASTLAVSSLSHA | Periplasm |  |
| >YP_026281.1 | *yjdP* | Uncharacterized protein YjdP | MKRFPLFLLFTLLTLSTVPAQA | Periplasm | Y |
| >YP_026283.1 | *ecnA* | Entericidin A | MMKRLIVLVLLASTLLTGC | Membrane | Y |
| >YP_588473.1 | *yifL* | Uncharacterized lipoprotein YifL | MKNVFKALTVLLTLFSLTGC | Membrane |  |

Table S1. *E. coli* proteins with known or predicted signal peptides. Protein sequences encoded by *E. coli* MG1655 were retrieved from Uniprot ([www.uniprot.org](http://www.uniprot.org)) and analyzed for the presence of a signal peptide using the SignalP 4.1 server (<http://www.cbs.dtu.dk/services/SignalP>). The n-regions were analyzed manually; twin arginines are colored red, twin lysines green, lys-arg or arg-lys are shown in blue. Pairings of Arg-Asn, Arg-Asp, Arg-Gln, Arg-Glu and Arg-His (which from this study can be suppressed by increased signal peptide hydrophobicity) are shown in orange. For those signal peptides harboring any of these dipeptides in their n-regions the approximate location of the h-region is shown in underline. The start of the h-region was defined by phobius (<http://phobius.sbc.su.se/>), the end of the h-region and the cleavage site was defined by signalP. Pairings within five residues of the start of the h-region were considered likely to interact with the Tat pathway. Those marked as N? have one of the indicated pairings 5 or more residues away from the h-region and are considered unlikely to interact with the Tat pathway. Known or probable Tat substrates are shown in yellow highlight; EfeO is predicted to be a Tat substrate but this has not been demonstrated experimentally.
