# Supplementary material for: Signal Peptide Hydrophobicity Modulates Interaction with the Twin-Arginine Translocase
Source: mBio. 2017 Aug 1;8(4):e00909-17. doi: 10.1128/mBio.00909-17 (PMC5539426; doi:10.1128/mBio.00909-17)
Supplement: TABLE S2 [file mbo004173403st2.docx]

| Plasmid | Description | Reference |
| --- | --- | --- |
| pTAT101 | Low copy number vector producing TatABC under the control of *tat* promoter. Kan^r^ | (37) |
| pTH19kr | Low copy-number cloning vector. Backbone of pTAT101 | (65) |
| pTAT101CP48L | As pTAT101, TatC P48L exchange | (18) |
| pTAT101CF94D | As pTAT101, TatC F94D exchange | (27) |
| pTAT101C103K | As pTAT101, TatC E103K exchange | (37) |
| pTAT101CV145E | As pTAT101, TatC V145E exchange | (18) |
| pTAT101CQ215R | As pTAT101, TatC Q215R exchange | (37) |
| pSU18 | Medium copy vector. Cm^r^ | (66) |
| pSUSufIss-mAmiA | pSU18, carrying SufIss-mAmiA | (27) |
| pSUSufIssA11L-mAmiA | As pSUSufIss-mAmiA, SufIss A11L exchange | This work |
| pSUSufIssS12L-mAmiA | As pSUSufIss-mAmiA, SufIss S12L exchange | This work |
| pSUSufIssG13L-mAmiA | As pSUSufIss-mAmiA, SufIss G13L exchange | This work |
| pSUSufIssA15L-mAmiA | As pSUSufIss-mAmiA, SufIss A15L exchange | This work |
| pSUSufIssA18L-mAmiA | As pSUSufIss-mAmiA, SufIss A18L exchange | This work |
| pSUSufIssG19L-mAmiA | As pSUSufIss-mAmiA, SufIss G19L exchange | This work |
| pSUSufIssA20L-mAmiA | As pSUSufIss-mAmiA, SufIss A20L exchange | This work |
| pSUSufIssRD-mAmiA | As pSUSufIss-mAmiA, SufIss R6D exchange | This work |
| pSUSufIssREmAmiA | As pSUSufIss-mAmiA, SufIss R6E exchange | This work |
| pSUSufIssRH-mAmiA | As pSUSufIss-mAmiA, SufIss R6H exchange | This work |
| pSUSufIssRNmAmiA | As pSUSufIss-mAmiA, SufIss R6N exchange | This work |
| pSUSufIssRQ-mAmiA | As pSUSufIss-mAmiA, SufIss R6Q exchange | This work |
| pSUSufIssKHmAmiA | As pSUSufIss-mAmiA, SufIss R5K, R6H exchange | This work |
| pSUSufIssKQ-mAmiA | As pSUSufIss-mAmiA, SufIss R5K, R6Q exchange | This work |
| pSUSufIssKKmAmiA | As pSUSufIss-mAmiA, SufIss R5K, R6K exchange | This work |
| pSUSufIssHHmAmiA | As pSUSufIss-mAmiA, SufIss R5H, R6H exchange | This work |
| pSUSufIssRD-S12L -mAmiA | As pSUSufIssRD-mAmiA, SufIss S12L exchange | This work |
| pSUSufIssRE-S12L mAmiA | As pSUSufIssRE-mAmiA, SufIss S12L exchange | This work |
| pSUSufIssRH-S12L -mAmiA | As pSUSufIssRH-mAmiA, SufIss S12L exchange | This work |
| pSUSufIssRN-S12L mAmiA | As pSUSufIssRN-mAmiA, SufIss S12L exchange | This work |
| pSUSufIssRQ-S12L -mAmiA | As pSUSufIssRQ-mAmiA, SufIss S12L exchange | This work |
| pSUSufIssKH-S12L mAmiA | As pSUSufIssKH-mAmiA, SufIss S12L exchange | This work |
| pSUSufIssKQ-S12L -mAmiA | As pSUSufIssKQ-mAmiA, SufIss S12L exchange | This work |
| pSUSufIssKK-S12L mAmiA | As pSUSufIssKK-mAmiA, SufIss S12L exchange | This work |
| pSUSufIssHH-S12L mAmiA | As pSUSufIssHH-mAmiA, SufIss S12L exchange | This work |
| pSUSufIssKK-G13L mAmiA | As pSUSufIssKK-mAmiA, SufIss G13L exchange | This work |
| pSUSufIssKK-A15L mAmiA | As pSUSufIssKK-mAmiA, SufIss A15L exchange | This work |
| pSUSufIssKK-G19L mAmiA | As pSUSufIssKK-mAmiA, SufIss G19L exchange | This work |
| pSUSufIssS12LG13L-mAmiA | As pSUSufIss-mAmiA, SufIss S12L, G13L exchange | This work |
| pSUSufIssS12LG13L4L15L-mAmiA | As pSUSufIss-mAmiA, SufIss S12L, G13L, I14L, A15L exchange | This work |
| pSUSufIssS17LS18L19L20L-mAmiA | As pSUSufIss-mAmiA, SufIss C17L, A18L, G19L, A20L exchange | This work |
| pSUDsbAss-mAmiA | As pSUSufIss-mAmiA, *sufIss* substituted with *dsbAss* | This work |
| pSUDsbAssi16K-mAmiA | As pSUDsbAss-mAmiA, DsbAss 16K insertion | This work |
| pSUOmpAss-mAmiA | As pSUSufIss-mAmiA, *sufIss* substituted with *ompAss* | This work |
| pSUOmpAssi18K-mAmiA | As pSUOmpAss-mAmiA, OmpAss 18K insertion | This work |
| pQE80-SufIhis | pQE80 carrying *sufI*_his_ | (27) |
| pQE80-SufIhis-S12L | As pQE80-SufIhis, SufI S12L exchange | This work |
| pQE80-SufIhis-G13L | As pQE80-SufIhis, SufI S13L exchange | This work |
| pQE80-SufIhis-A15L | As pQE80-SufIhis, SufI S15L exchange | This work |
| pQE80-SufIhis-G19L | As pQE80-SufIhis, SufI S19L exchange | This work |
| pQE80-SufIhis-S12LG13L | As pQE80-SufIhis, SufI S12L, G13L exchange | This work |
| pQE80-SufIhis-12L13L14L15L | As pQE80-SufIhis, SufI S12L, G13L, I14L, A15L exchange | This work |
| pQE80-SufIhis- 17L18L19L20L | As pQE80-SufIhis, SufI C17L, A18L, G19L, A20L exchange | This work |
| pFAT75ΔA -SufIhis | *tatBC* with *sufI_his_* in pQE60 | (18) |
| pFAT75ΔA-SufIFLAG | As pFAT75ΔA-SufIhis, *sufIhis* substituted with *sufI*FLAG | This work |
| pFATBChis-SufIFLAG | As pFAT75ΔA-SufIFLAG, *tatC* his-tagged | This work |
| pFATBChis-SufIS12LFLAG | As pFATBChis-SufIFLAG, SufI S12L exchange | This work |
| pFATBChis-SufIG13LFLAG | As pFATBChis-SufIFLAG, SufI G13L exchange | This work |
| pFATBChis-SufIA15LFLAG | As pFATBChis-SufIFLAG, SufI A15L exchange | This work |
| pFATBChis-SufIG19LFLAG | As pFATBChis-SufIFLAG, SufI G19L exchange | This work |
| pFATBC94Dhis-SufIFLAG | As pFATBChis-SufIFLAG, TatC F94D exchange | This work |
| pFATBC94Dhis-SufIS12LFLAG | As pFATBChis-SufIS12LFLAG, TatC F94D exchange | This work |
| pFATBC94Dhis-SufIG13LFLAG | As pFATBChis-SufIG13LFLAG, TatC F94D exchange | This work |
| pFATBC94Dhis-SufIA15LFLAG | As pFATBChis-SufIA15LFLAG, TatC F94D exchange | This work |
| pFATBC94Dhis-SufIG19LFLAG | As pFATBChis-SufIG19LFLAG, TatC F94D exchange | This work |
| pFATBC94Dhis-SufI12L13L14L15LFLAG | As pFATBChis-SufIFLAG, SufI S12L, G13L, I14L, A15L exchange | This work |
| pFATBC94Dhis-SufI17L18L19L20LFLAG | As pFATBChis-SufIFLAG, SufI C17L, A18L, G19L, A20L exchange | This work |
| pFATBChis-OmpAssi18KSufIFLAG | As pFATBChis-OmpAssSufIFLAG, OmpA 18K insertion | This work |
| pQEBChis-OmpAFLAG | pQE80 coproducing TatBChis and OmpAFLAG | This work |
| pQEBChis-DsbAFLAG | pQE80 coproducing TatBChis and DsbAFLAG | This work |

**Table S2.** Plasmids used and constructed in this study.
