# Supplementary material for: Signal Peptide Hydrophobicity Modulates Interaction with the Twin-Arginine Translocase
Source: mBio. 2017 Aug 1;8(4):e00909-17. doi: 10.1128/mBio.00909-17 (PMC5539426; doi:10.1128/mBio.00909-17)
Supplement: TABLE S3 [file mbo004173403st3.docx]

| SufIA11L-F | GTCAGTTCATTCAGCTGTCGGGGATTGCAC |
| --- | --- |
| SufIA11L-R | GTGCAATCCCCGACAGCTGAATGAACTGAC |
| SufI S12L-F | GTTCATTCAGGCACTGGGGATTGCACTTTG |
| SufI S12L-R | CAAAGTGCAATCCCCAGTGCCTGAATGAAC |
| SufIG13L-F | GTTCATTCAGGCATCGCTGATTGCACTTTGTGC |
| SufIG13L-R | GCACAAAGTGCAATCAGCGATGCCTGAATGAAC |
| SufIA15L-F | CAGGCATCGGGGATTCTGCTTTGTGCAGGCGC |
| SufIA15L-R | GCGCCTGCACAAAGCAGAATCCCCGATGCCTG |
| SufIA18L-F | GGGATTGCACTTTGTCTGGGCGCTGTTCCCCTG |
| SufIA18L-R | CAGGGGAACAGCGCCCAGACAAAGTGCAATCCC |
| SufIG19L-F | GATTGCACTTTGTGCACTGGCTGTTCCCCTGAAG |
| SufIG19L-R | CTTCAGGGGAACAGCCAGTGCACAAAGTGCAATC |
| SufIA20L-F | GCACTTTGTGCAGGCCTGGTTCCCCTGAAGGCC |
| SufIA20L-R | GGCCTTCAGGGGAACCAGGCCTGCACAAAGTGC |
| SufI S12LG13L-F | GTTCATTCAGGCACTGCTGATTGCACTTTGTGC |
| SufI S12LG13L-R | GCACAAAGTGCAATCAGCAGTGCCTGAATGAAC |
| SufI S12LG13L14L15L-F | CATTCAGGCACTGCTGCTGCTGCTTTGTGCAGGCGCTG |
| SufI S12LG13L14L15L-R | CAGCGCCTGCACAAAGCAGCAGCAGCAGTGCCTGAATG |
| SufI17L18L19L20L-F | GCATCGGGGATTGCACTTCTGCTGCTGCTGGTTCCCCTGAAGGCCAGC |
| SufI17L18L19L20L-R | GCTGGCCTTCAGGGGAACCAGCAGCAGCAGAAGTGCAATCCCCGATGC |
| SufIF | ATGTCACTCAGTCGGCGTC |
| SufIR | TTACGGTACCGGATTGACC |
| SufIF8X1 | CAGTCGGCGTCAGNNNATTCAGGCATCGG |
| SufIF8X2 | CCGATGCCTGAATNNNCTGACGCCGACTG |
| FAT75TatChis-1 | GAAAGCGAAAAAACTGAAGAACATCACCATCACCATCACTAAGGGCCCCATTAAAGAG |
| FAT75TatChis-2 | CTCTTTAATGGGGCCCTTAGTGATGGTGATGGTGATGTTCTTCAGTTTTTTCGCTTTC |
| FAT75SufIFLAG-1 | GTTGGTCAATCCGGTACCGGATTACAAGGATGACGACGATAAGTAAGCTTAATTAGCTGAGCTTG |
| FAT75SufIFLAG-2 | CAAGCTCAGCTAATTAAGCTTACTTATCGTCGTCATCCTTGTAATCCGGTACCGGATTGACCAAC |
| DsbAss-FE | CCGGAATTCGTTTTACATGGAGCAAATATGAAAAAGATTTGGCTGGCG |
| DsbAss-R | GTTCGTCTTTCGCCGATGCGCTAAACGC |
| DsbAss16inK-R | GTTCGTCTTTCGCCGATGCGCTtttAAACGCTAAAACTAAAC |
| DsbA-mAmiA-F | AGCGCATCGGCGAAAGACGAACTTTTAAAAACC |
| OmpA-FE | CCGGAATTCGTTTTACATGGAGCAAATATGAAAAAGACAGCTATCGCG |
| OmpAss-R | GTTCGTCTTTGGCCTGCGCTACGGTAG |
| OmpA18inK-R | GTTCGTCTTTGGCCTGCGCTACTTTGGTAGCGAAACCAG |
| OmpA-mAmiA-F | GTAGCGCAGGCCAAAGACGAACTTTTAAAAACC |
| amiA-mRX | GAC TCT AGA TTA TCG CTT TTT C |
| FATHF-DsbA-F | CACCATCACTAAGGGCCCCATTAAAGAGGAGAAATTAACCATGAAAAAGATTTGGCTGGCG |
| FATHF-OmpA-F | CACCATCACTAAGGGCCCCATTAAAGAGGAGAAATTAACCATGAAAAAGACAGCTATCGCG |
| FATHF-OmpA18K-R | CAAGTAGCGGCGGAACGGGTAGCGGTTGCTGTTGCCCGGCGGCCTGCGCTACTTTGGTAG |
| OmpAFLAG-SR | ACGCGTCGACTTACTTATCGTCGTCATCCTTGTAATCAGCCTGCGGCTGAGTTAC |
| DsbAFLAG-SR | ACGCGTCGACTTACTTATCGTCGTCATCCTTGTAATCTTTTTTCTCGGACAGATATTTCAC |
| QEF | CCCGAAAAGTGCCACCTG |

**Table S3.** Oligonucleotides used in this study.
